# A Novel Human Cellular System for Studying Normal Aging and for Anti‐Aging Discovery

**DOI:** 10.1111/acel.70352

**Published:** 2026-01-20

**Authors:** Zhen Feng, Cheuk Shuen Li, Haifeng Fu, Wenxin Jiang, Weiyu Zhang, Yingzhang Huang, Yunying Huang, Timothy Theodore Ka Ki Tam, Yang Li, Fang Liu, Liming Lu, Yin Lau Lee, William Shu Biu Yeung, Gordon Dougan, Pentao Liu

**Affiliations:** ^1^ InnoHK Centre for Translational Stem Cell Biology Science Park Hong Kong; ^2^ Shenzhen Key Laboratory of Fertility Regulation, Reproductive Medicine Centre The University of Hong Kong—Shenzhen Hospital Shenzhen China; ^3^ School of Biomedical Sciences, Li Ka Shing Faculty of Medicine The University of Hong Kong Hong Kong China; ^4^ Shanghai Institute of Immunology Shanghai Jiao Tong University School of Medicine Shanghai China; ^5^ Department of Obstetrics and Gynaecology, School of Clinical Medicine, Li Ka Shing Faculty of Medicine The University of Hong Kong Hong Kong China; ^6^ Department of Medicine University of Cambridge Cambridge UK

## Abstract

Aging studies using animal and cellular models have uncovered key proteins and pathways central to organismal aging. However, these models differ genetically and physiologically from human aging, posing challenges in translating discoveries to human contexts. In this study, we present a human normal cell aging model based on the development of cytotrophoblasts (CTBs) to syncytiotrophoblasts (STBs) in the placenta. The in vitro‐derived STBs from human trophoblast stem cells (hTSCs) recapitulate the maturation and major cellular aging features of in vivo CTB‐STB, including multinucleation, hormone secretion, cell cycle arrest, genome instability, epigenetic changes, activation of endogenous transposable elements, and senescence‐associated secretory phenotypes (SASPs). Notably, the progressive senescence in the trophoblast system closely matches the predicted aging trajectory of other human tissue stem cells. Known anti‐aging molecules, such as mTOR inhibitors and senolytics, attenuate senescence signals in STBs. The established CGA‐EGFP reporter hTSC line enables scalable and quantitative screening and identified candidates with it can be further extended to other context‐specific aging processes like that of skin fibroblasts. The hTSC‐STB system represents a novel physiologically accelerated cellular aging model, bridges the gap between fundamental aging research and interventions, and prioritizes anti‐aging candidates for clinical development.

## Graphical abstract

This study introduces a human cellular aging model using placental trophoblasts (hTSC‐STBs) that mimics key aging features like senescence and genomic instability. It aligns with human tissue aging and responds to anti‐aging treatments, offering a scalable platform to screen potential therapies and bridge lab findings to clinical applications.

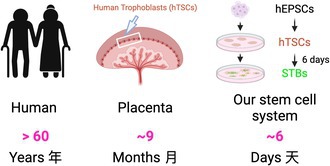

## 1 Introduction

Organism aging is driven by interconnected processes, including cellular senescence, genomic instability, chronic inflammation, etc. (Lopez‐Otin et al. 2023). Cellular senescence, a state where cells permanently stop dividing but remain metabolically active, is essential for cancer prevention early in life (Storer et al. 2013; Munoz‐Espin et al. 2013) but a key driver of aging when dysregulated (Lopez‐Otin et al. 2023). Senescent cells develop genomic instability due to unrepaired mutations, chromosomal rearrangements, and micronuclei formation. The derepression of transposable elements, notably long interspersed nuclear element‐1 (LINE‐1) and human endogenous retrovirus K (HERVK), contributes to genomic instability with insertional mutations and activates the cGAS/STING and TLR pathways (Decout et al. 2021; Lima‐Junior et al. 2021; Sun et al. 2013). These signaling, along with mTOR, TNF, and JAK–STAT activation (Huang et al. 2022), drive the production of the senescence‐associated secretory phenotypes (SASPs), a collection of pro‐inflammatory cytokines (Decout et al. 2021; de Magalhaes 2024; Chaib et al. 2022). The SASPs propagate senescence to neighboring cells, inducing “inflammaging” and accelerating tissue dysfunction and organ aging (Acosta et al. 2013; Moiseeva et al. 2023).

Advances in aging mechanisms and models have revolutionized the field of longevity, age‐related diseases, and potential interventions. Model organisms like yeast, *C. elegans* , *D. melanogaster* , and mice are used to investigate aging mechanisms, leading to the discovery of conserved proteins and pathways (Lopez‐Otin et al. 2023; Fabrizio et al. 2001; Kenyon et al. 1993; Tatar et al. 2001; Longo et al. 2012; Di Francesco et al. 2024). Studies on replicative and chronological lifespan in yeast have uncovered roles for sirtuins and mitochondrial function in aging (Longo et al. 2012). Nutrient‐sensing pathways, such as the insulin/IGF‐1 signaling (IIS) pathway and mTOR pathway, are well‐documented in modulating lifespan across species (Kenyon et al. 1993; Tatar et al. 2001; Di Francesco et al. 2024). Transgenic mouse models and calorie restriction studies have provided critical insights into mammalian aging (Di Francesco et al. 2024).

Programmed cellular senescence also occurs in the short‐lived placenta (Cox and Redman 2017; Gal et al. 2020). It is a double‐edged sword. Physiological senescence supports placental maturation and labor onset (Gal et al. 2020). Senescent cells in the placenta secrete SASPs such as matrix metalloproteinases (MMPs) and cytokines (e.g., IL‐6, IL‐8), which remodel spiral arteries and aid tissue repair. Term placenta exhibits increased senescence markers (p16, p21, SA‐β‐gal) as a normal programmed process to initiate labor. SASPs at term (e.g., TGF‐β, PAI‐1) promote membrane rupture and uterine contractions via collagen degradation and prostaglandin activation. Pathologically, abnormal placental senescence underlies preeclampsia, IUGR, and preterm birth (Kajdy et al. 2023). Excessive senescence leads to placental ischemia and oxidative stress. High placental p16ᴵᴺᴷ^4^ᵃ and circulating sFlt‐1/PlGF ratio predict preeclampsia (Sugulle et al. 2024). Premature senescence reduces trophoblast proliferation, resulting in a smaller, dysfunctional placenta associated with mitochondrial dysfunction, telomere shortening, and low vascularization. Dysregulated placenta senescence causes premature breakdown of fetal membranes due to MMP‐9/TIMP‐1 imbalance. Future directions, like targeting placental senescence with precision therapies (e.g., nanoparticle‐delivered senolytics), might improve pregnancy outcomes.

Recent studies suggest a functional link between the placenta and female aging, as pregnancy has been reported to accelerate aging, followed by a postpartum recovery (Pham et al. 2024; Ryan et al. 2024). Additionally, placenta‐specific genes, such as PSGs and PAPPA, have been detected at high levels in aged cells and the plasma of aged individuals (Liu et al. 2022; Bi et al. 2024). The key placental cell type, cytotrophoblast (CTB), can differentiate into multinucleated syncytiotrophoblast (STB) and extravillous trophoblast (EVT) (Knofler et al. 2019). The in vivo CTB‐STB system can now be recapitulated in vitro, where human trophoblast stem cells (hTSCs) mature to STBs within a week (Okae et al. 2018; Gao et al. 2019; Ruan et al. 2022). Studies have reported partial senescence features in mature in vivo STBs, including elevated levels of cell cycle inhibitors, SASPs, and telomere shortening (Cox and Redman 2017; Gal et al. 2020; Sultana et al. 2017), but these findings lacked systematic characterization.

This study comprehensively investigates senescence signatures during both in vivo and in vitro STB development and, for the first time, explores their potential applications. We demonstrated that the hTSC‐STB system accurately mimics the natural aging of in vivo STBs and recapitulates senescence hallmarks, including declines in DNA damage repair, genomic instability, epigenetic alterations, stem cell exhaustion, telomere and mitochondria dysfunction, SASPs, and impaired nutrient‐sensing network. Many interventions extend lifespan in classical aging models but show limited efficacy in humans due to species‐specific biology. We found that both the in vivo CTB‐STB and in vitro hTSC‐STB systems can represent the physiologically relevant human aging process in general, evidenced by a strong correlation with predicted donor age of muscle stem cells (MuSCs) and hematopoietic stem cells (HSCs). This accelerated human cellular aging model offers a rapid approach to evaluating the anti‐aging activities of known molecules, such as mTOR inhibitors and senolytics. We established a reporter system for high‐content screening and identified novel anti‐aging molecules that could be applied to human dermal fibroblast aging. We anticipate that at a transformative juncture of aging research, our hTSC‐STB model will help bridge the gap between laboratory discoveries and human applications, complementing other emerging approaches such as iPSC, organoids, and computational and AI‐driven models (Pitrez et al. 2024; Nature Aging 2023; Kalyakulina et al. 2024).

## 2 Results

### 2.1 Human Placenta Trophoblasts Exhibit Major Senescence Hallmarks

**FIGURE 1:**
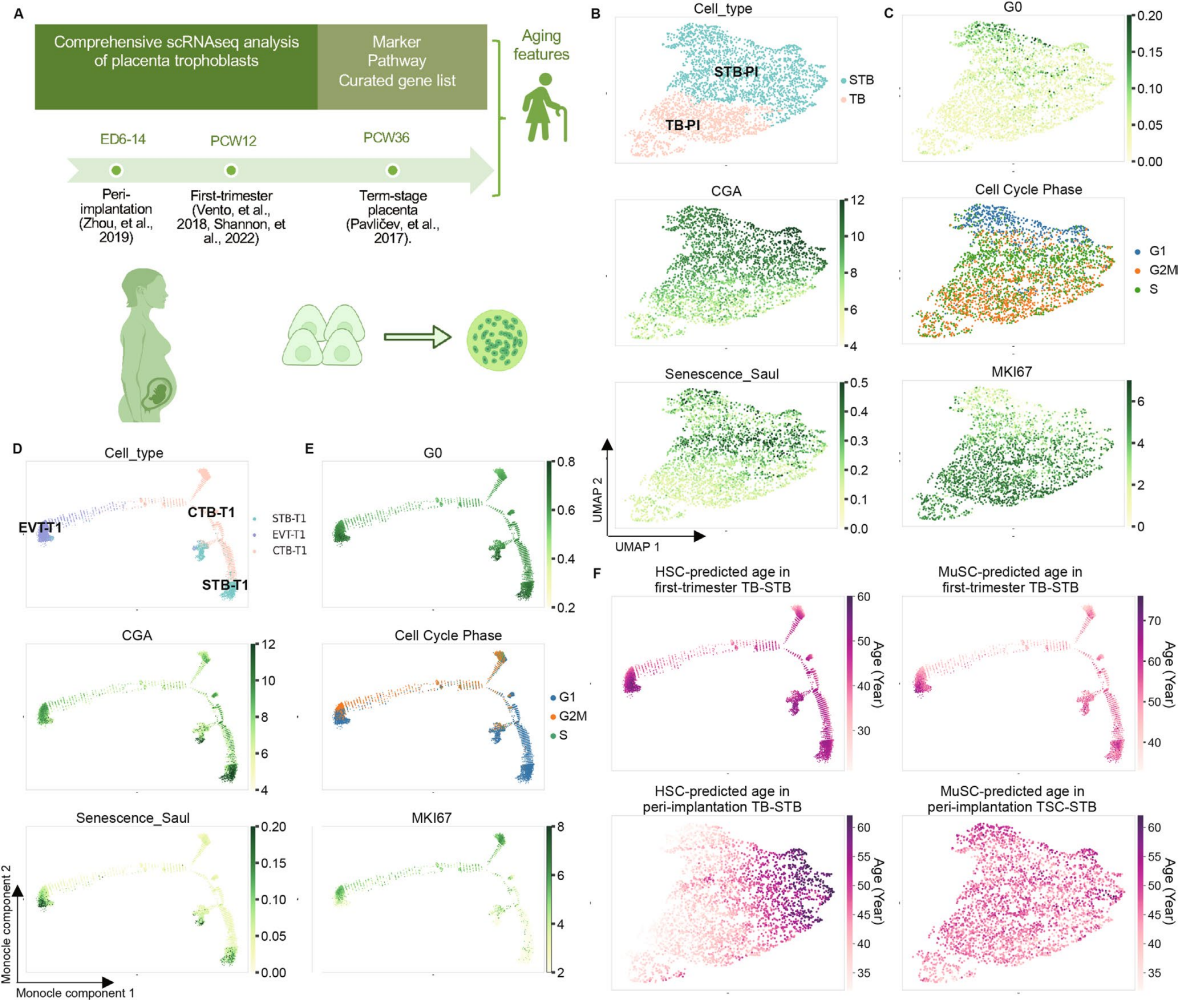
Syncytiotrophoblast development in vivo expresses senescence hallmarks. (A) Schematic diagram showing the comprehensive scope of the bioinformatic analyses of in vivo trophoblast development of different pregnancy stages for molecular‐level aging features. Curated aging hallmarks are reflected by different pathway scores and/or marker expression levels; (B, C) The uniform manifold approximation and projection (UMAP) analysis displayed increased STB marker gene CGA and senescence features along the differentiation trajectory from late trophectoderm and trophoblast progenitors (TB) to syncytiotrophoblast (STB) subset from the peri‐implantation stage (3238 cells, Zhou et al. 2019). Cell type was annotated by Castel (2020). PI, peri‐implantation. Senescence signature scores were computed by a weighted average senescence gene list from a curated study (*Senescence_Saul*). The colors suggest a shift in the cell cycle phases, including an increase in G0 level (upper in C), a switch from G2M/S‐phase to G1 (middle), and reduced proliferation marker Ki67 (MKI67) expression (bottom); (D, E) Similar to (B, C), increased STB marker CGA and senescence features along the Monocle differentiation trajectory from placenta villous trophoblast (CTB) to syncytiotrophoblast cell (STB) and extravillous trophoblast (EVT) during the first‐trimester stage (13,777 cells, Vento‐Tormo et al. 2018). Cell types specified in the original paper have been visualized. T1: First trimester. Gene expression levels were visualized in log‐normalized values for all plots hereafter; (F) The predicted age in the STB differentiation process in the peri‐implantation stage (lower) and first‐trimester stage (upper), respectively, where each cell identification was the same as in (B) and (D). Linear regression models were fitted to HSC data and MuSC data with corresponding chronological ages for the prediction (Methods). Color scale: Deeper color indicates higher predicted age (in years).

The trophoblast development of CTBs to STBs in healthy placenta exhibits aging features, including an increase in senescence‐associated beta‐galactosidase (SA‐β‐gal) staining, high levels of cyclin kinase inhibitors and mTOR, telomeres attrition, and chronic inflammation (Cox and Redman 2017; Ciampa et al. 2023; Gal et al. 2019; Velicky et al. 2018). These data collectively position the placenta as a potential model for investigating human aging. The published aging phenotype of trophoblast was primarily identified at the relatively mature placenta (Cox and Redman 2017; Gal et al. 2019; Qi et al. 2022). Here, we systematically profiled cellular aging signatures in CTB‐STB development at single‐cell resolution, focusing on the early placental stage (Figure 1A). We re‐analyzed published single‐cell RNA sequencing (scRNAseq) data in various placental stages, encompassing peri‐implantation (refer to PI, 4139 cells from Zhou et al. (2019), accession GSE109555, Figure 1B,C), first‐trimester (refer to T1, 13,777 cells from Vento‐Tormo et al. (2018) in Figures 1D,E and S1A, accession E‐MTAB‐6701, 3854 cells from Shannon et al. (2022) in Figure S1B,C, accession GSE174481), and term‐stage placenta (averaged count for CTB of 3 stages and STB from Pavlicev et al. (2017) in Figure S1D, accession GSE87720). Trajectory analyses on reduced dimensions revealed the primary development paths from CTBs into STBs and EVTs (Figures 1B,D and S1A,B).

We first cross‐examined a curated high‐fidelity senescence gene set (refer to Saul et al. 2022, Table S1), which was generated by an in‐depth literature search and validated in human biopsies, along the trophoblast differentiation. The senescence scores progressively increased during STB development across all stages of pregnancy (Figures 1B,D and S1B,D). This was paralleled by a shift from the G2M/S phase in progenitor cells to G1/G0 arrest in STBs, indicating cell cycle arrest and a transition to a senescent state (Figures 1C,E and S1C,D). Cellular senescence markers, including CDK inhibitor expression and low expression of proliferation marker Ki67 (MKI67), could be observed in the STBs across the stages (Figures 1C,E and S1A,C).

We particularly deconvoluted the well‐documented aging hallmarks within the scRNAseq data of the early placental stages in detail (Data of T1 showed here, PI not shown). Using comprehensive gene signature scores, STBs exhibited lower DNA repair gene expression compared to CTBs (Figure S1A), including base excision repair BER, nucleotide excision repair NER, non‐homologous end joining repair NHEJ, Fanconi anemia DNA repair Fanc, and homologous recombination repair HR. Lamin B1 (LMNB1), a marker of nuclear integrity and genomic stability (Lopez‐Otin et al. 2023; de Magalhaes 2024; Gil 2023), was also reduced in STBs (Figure S1A). Regarding histone modifications, STBs exhibited a trend toward decreased levels of H3K27me3 and H3K9me3, accompanied by declined methyltransferase EZH2 (regulate H3K27me3) and SUV39H1 (regulate H3K9me3) and increased KDM6B (also known as JMJD3, H3 lysine demethylase) (Figure S1A), which plays a central role in inflammation and heterochromatin alterations during aging (Lopez‐Otin et al. 2023; Fogarty et al. 2015; Salminen et al. 2014; Djeghloul et al. 2016). In addition, previously underexplored aging features in early trophoblasts (Cheung et al. 1999; Schaetzlein et al. 2004), telomere attrition, could be corroborated by a decrease in the expression of telomeric repeat‐binding factors (TERF1, TERF2; Figure S1A). Other aging‐related changes, including increased SASP secretion and metabolism alterations, were also observed in STBs (Figure S1A). In this analysis, EVTs have been similarly identified with senescence features (marked by damaged DNA repair, SASP, and metabolic shifts) (Figures 1D and S1A, Table 1). The scRNAseq revealed more prominent aging signatures (e.g., downregulated MKI67, CDK1, SUV39H1, and TERF1/2; LMNB1 loss) in the CTB‐to‐STB pathway (Figures 1E and S1A, Table 1), underscoring replicative decline, telomeres dysfunction, and genomic instability uniquely, prioritizing it for subsequent investigations.

**TABLE 1:**
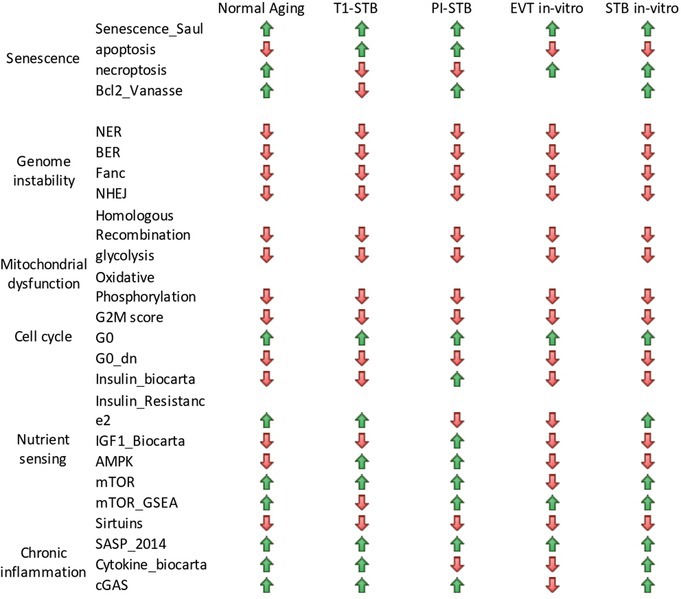
Known aging markers in the in vivo trophoblasts and the in vitro hTSCs‐STBs system.

*Note*: Change of Each Age‐Related Pathway for Differentiated Trophoblast State‐Subtype: 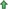: Increased from the cytotrophoblast/trophoblast stem cells of that stage, 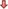: Decreased from the cytotrophoblast/trophoblast stem cells of that stage.

All tissue‐specific stem cells, such as muscle stem cells (MuSCs) (Kedlian et al. 2024) and hematopoietic stem cells (HSCs) (Lee et al. 2023), undergo aging. To assess the relevance of the CTB‐STB development to cellular aging beyond the placenta, we applied a linear regression model to simplistically perform global‐profile cross‐comparisons with MuSCs and HSCs. First, we trained the model on the whole transcriptome from 1902 MuSCs and 3568 (should be same as methods 4.23) HSCs from donors of various ages, spanning from young to old (Kedlian et al. 2024; Lee et al. 2023) (Figure S1E). Remarkably, the predicted donor ages of MuSCs and HSCs showed a consistent correlation with the CTB to STB development across the placental stages (PI and T1, Figure 1F). In contrast, MuSCs and HSCs themselves did not exhibit a strong correlation during aging (data not shown). Cellular aging features observed in other tissue contexts can, therefore, be effectively modeled by the CTB‐STB system. These results highlight the potential to extend insights from the CTB‐STB development to the aging processes of other systems, offering new perspectives on organismal aging in general.

### 2.2 The In Vitro Development of hTSCs Into STBs Mirrors a High Senescence Score Accompanied by Cell Cycle Arrest

Our re‐analysis of placental trophoblast scRNAseq data revealed key aging hallmarks, prompting us to establish an in vitro hTSC‐to‐STB development system that mirrors native trophoblast aging. The model will overcome the limitations of in vivo studies, such as scarce tissue access, ethical barriers, and static sampling.

**FIGURE 2:**
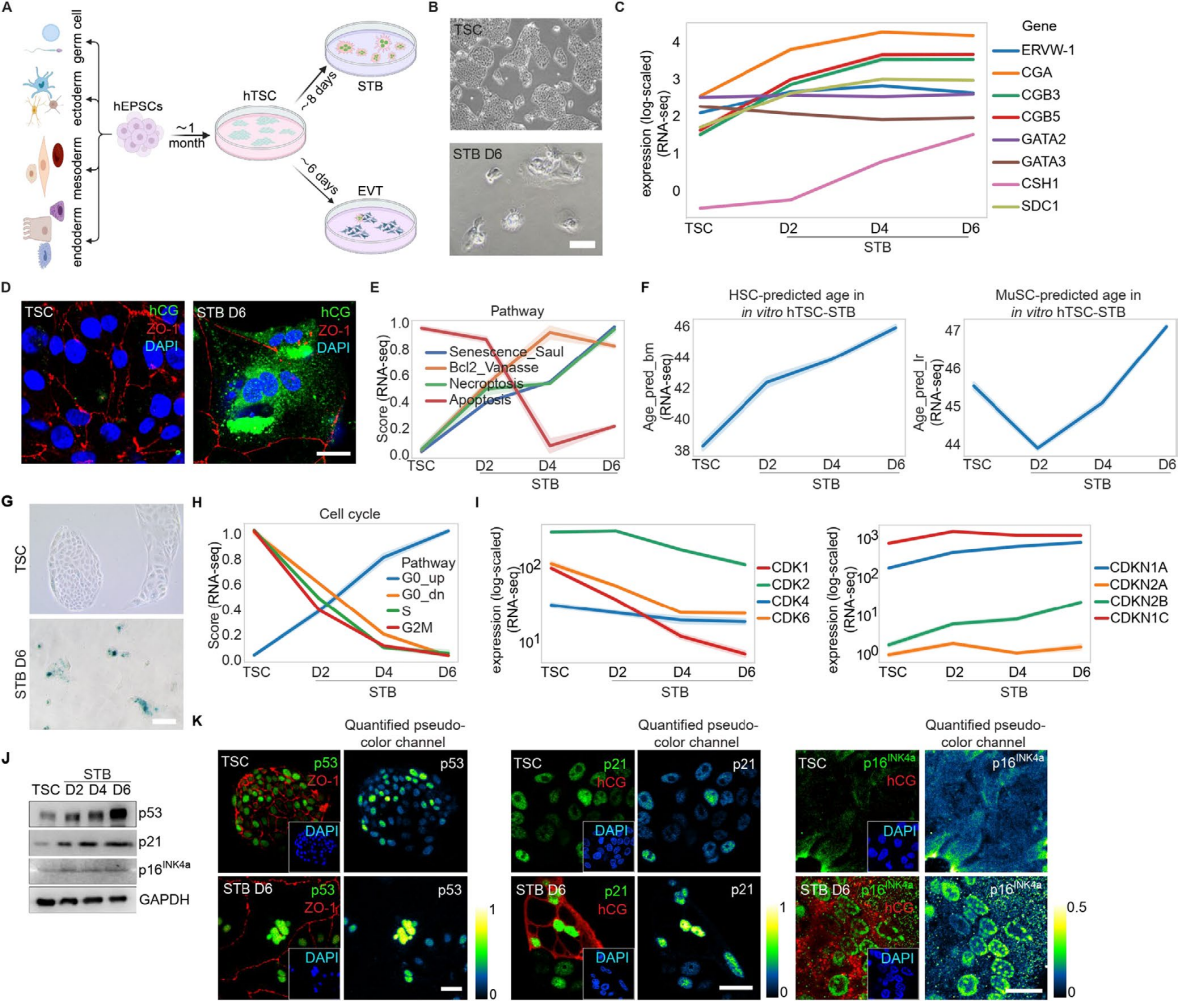
The development of hTSCs to STBs in vitro showed increased senescence features with cell cycle arrest, similar to in vivo STBs. (A) Schematic diagram of the establishment of hTSCs from hEPSCs and subsequent development to STBs and EVTs; (B) Morphology of hTSC cells and the differentiated fused villous STBs (D6); (C) RNA‐seq detection of expression of CGA, CGB3, CGB5, SDC1, CSH1, ERVW1, GATA2, and GATA3 genes in hTSCs and STBs on D2, D4, D6; (D) The representative immunofluorescence (IF) staining image of hCG together with tight junction‐associated scaffolding protein ZO‐1 in hTSCs and STBs showing much higher hCG expression in STBs; (E) The global senescence and necroptosis score increased dramatically across time points from hTSCs to STBs, while the apoptosis score was low, accompanied by high anti‐apoptotic proteins in the Bcl2 family; (F) The correlation of the predicted age from hTSCs to STBs with HSC and MuSC aging process; (G) The images of β‐gal staining in hTSC and STB cells; (H) Line chart showing scores of cell cycle, G0_up (up), G0_dn (down), S, and G2M across the hTSCs to STBs development process; (I) Bulk RNA‐seq analysis of expression level of CDK genes (CDK1, CDK2, CDK4, CDK6) and CDKIs genes (CDKN1A (p21), CDKN2A (p16), CDKN2B (p15), CDKN1C (p57/KIP2)) across time points from hTSCs to STBs; (J) Western blot for p53, p21, and p16^INK4a^ in hTSCs to STBs. The cell cycle‐related molecules (p53, p21, and p16^INK4a^) were much higher in STBs; (K) Representative IF staining images of p53, p21, and p16^INK4a^ together with ZO‐1 or hCG in hTSCs and STB D6 cells. The left merged images showed stained targeted molecules with a DAPI‐indicated nucleus image located at the bottom right corner; the right was quantified pseudocolor images of the targeted molecule (for instance, p53) in hTSCs and STBs (D6), respectively. Images in the rest of the figures were organized in a similar way. All experiments have been independently repeated three times. Scale bars, 10 μm.

Under the published protocol (Okae et al. 2018; Ruan et al. 2022), hTSCs (hTSC‐M1 and hTSC‐em), which were derived from human expanded potential stem cells (M1 hEPSC and hEPSC‐em) (Gao et al. 2019; Chen et al. 2023) and highly similar to other reported hTSC lines molecularly, matured into multinucleated STBs with villi in approximately 6 days (Figure 2A,B). We collected cells on day 0 (D0, hTSC) and differentiated days (STB D2, STB D4, STB D6) for molecular, transcriptomic, and proteomics analysis (Mass spectrometry analysis (MS) also included STB D8 samples) (accession GSE190432, PXD071811, Table S2). The development of hTSCs to STBs was accompanied by high levels of mature STB markers (Figures 2C,D and S2A) and elevated secretion of human chorionic gonadotropin (hCG) (Figure S2B). Conversely, genes encoding trophoblast transcription factors GATA2 and GATA3 exhibit minimal changes (Figure 2C). Further analysis revealed strong transcriptomic parallels between the hTSC‐STB in vitro and those in vivo in the T1 and PI stages (Figure S2C).

Similar to in vivo CTB‐STB data analysis, the in vitro development of hTSCs to STBs exhibited a progressively higher senescence score (Figures 2E and S2D), referring to Saul et al. (2022) (Table S1). Increased apoptosis resistance (characterized by low apoptosis level and activation of anti‐apoptotic proteins in the Bcl2 family) and high necroptosis are well‐established features of cellular senescence (Lopez‐Otin et al. 2023; Di Micco et al. 2021). At the transcriptomic level, the transition from hTSCs to STBs showed a clear upward trend in necroptosis and apoptosis resistance (Figure 2E). Adopting the same regression model for cross‐comparisons, RNAseq data revealed that the development of hTSCs to STBs positively correlated with the aging trajectory observed in HSCs and MuSCs (Kedlian et al. 2024; Lee et al. 2023) (Figure 2F), consistent with in vivo STBs (Figure 1F).

We next experimentally interrogated cellular senescence in in vitro STBs. hTSCs could self‐renew robustly, as indicated by the healthy cell proliferation rate (Figure S2E). However, their proliferation capacity rapidly decreased, compromising stemness maintenance upon STB induction (Figure S2E). Notably, a greater number of SA‐β‐gal‐positive cells were seen in STBs (Figures 2G and S2F). Transcriptomically, the G2M score and S phase score both decreased during hTSC‐STB development (Figure 2H), along with the reduced cell cycle and DNA replication scores in the proteomics (Figure S2G). Permanent cell cycle arrest in STBs was further confirmed by the absence of Ki67 (Figure S2H,I) and proteomics‐validated reductions in proliferating cell nuclear antigen (PCNA) and minichromosome maintenance (MCM) (Jurikova et al. 2016) (Figure S2J).

Senescence is typically mediated by the tumor suppressors TP53 and CDKN2A/p16^INK4a^, and their downstream effectors CDKN1A/p21 and RB‐E2F, respectively (Gorgoulis et al. 2019). Along the hTSC‐STB development axis, the expression of cyclin‐dependent kinases (CDKs) gradually decreased, while CDK inhibitors (CDKN1A/p21, CDKN2A/p16^INK4a^, CDKN2B/p15, CDKN1C/p57kip2) exhibited an opposing trend (Figure 2I–K). The higher levels of CDK inhibitors and other negative regulators of the cell cycle, such as p38MAPK (Anerillas et al. 2023) and p27 (Bastians et al. 1998), were further supported by western blotting and immunostaining (Figures 2J,K and S2K,L). Altogether, the accumulated senescence score and evidence of cell cycle arrest in STBs indicate the potential of the hTSC‐STB system to serve as an in vitro cellular aging model.

### 2.3 DNA Damage Repair Decline, Genome Instability, and Epigenetic Alterations in STBs

**FIGURE 3:**
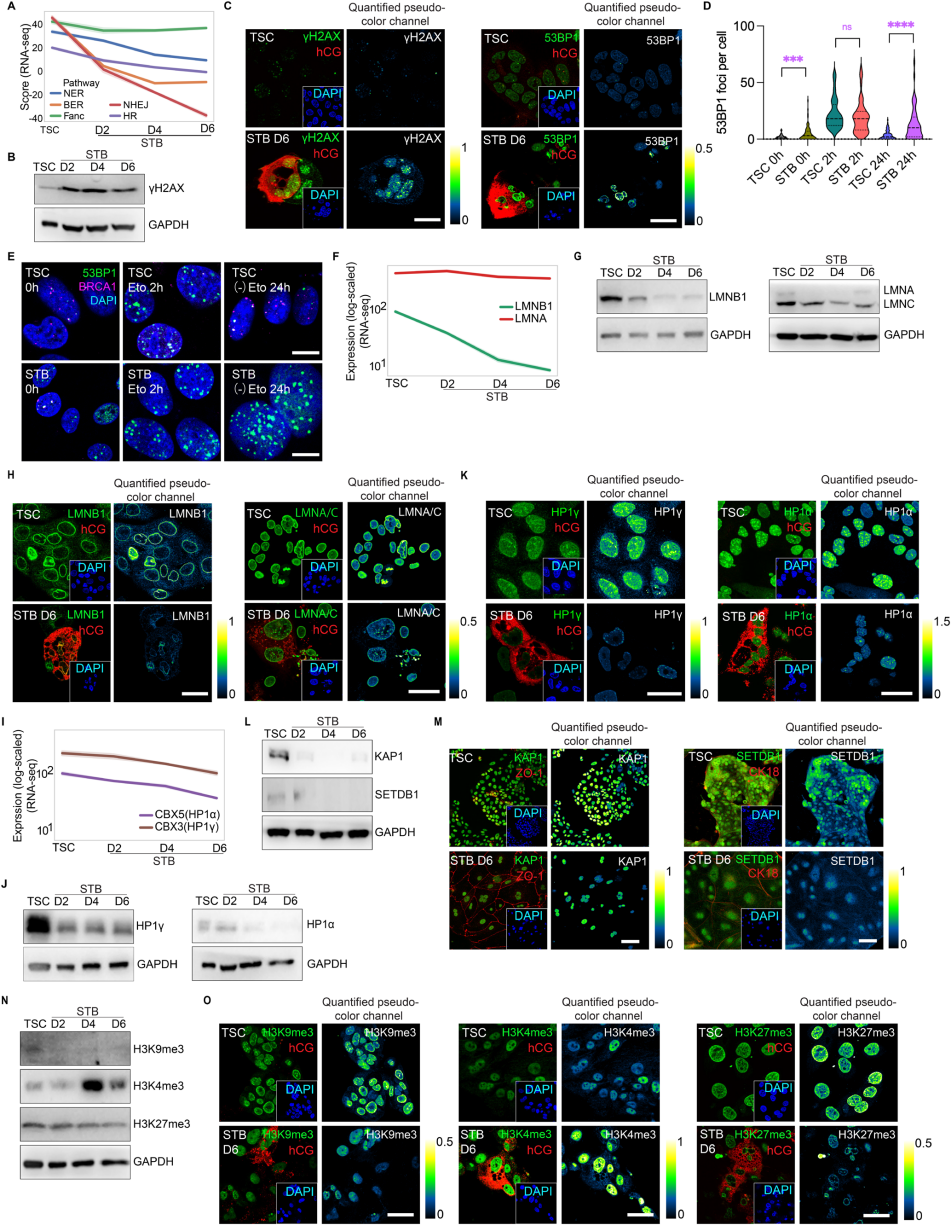
DNA repair decline, genome instability, and epigenetic alterations in the mature STBs in vitro. (A) Bulk RNA‐seq data analysis revealed decreased damaged DNA repair pathways (NER, BER, NHEJ, Fanc, HR) in STBs; (B) Western blot detected increased DNA damage marker γH2AX along hTSCs development into STBs (D2, D4, D6); (C) Representative immunostaining images of γH2AX and 53BP1 in hTSCs and STBs (D6); (D) Quantification of 53BP1 foci per cell in hTSCs and STBs at 0 h, Eto treatment 2 h, and 24 h recover after removing Eto treatment ((−)Eto); (E) Representative immunostaining images of 53BP1 and BRCA1 in hTSCs and STBs at 0 h, Eto 2 h, and (−)Eto 24 h timepoints; (F) Line chart showing decreasing genome instability associated markers lamins (LMNA and LMNB1) in the transcriptome of hTSCs and STBs in vitro; (G) Western blot of LMNB1 and LMNA/C levels in hTSCs and STBs; (H) Representative immunostaining images of LMNB1 and LMNA/C in hTSCs and STBs (D6); (I) Line chart shows decreasing of HP1 (HP1α and HP1γ) in the transcriptome of hTSCs and STBs in vitro; (J) Western blot of HP1α and HP1γ levels in hTSCs and STBs (D2, D4, D6); (K) Representative immunostaining images of HP1α and HP1γ in hTSCs and STBs (D6); (L) Western blot to detect KAP1 and SETDB1 in hTSCs and STBs on D2, D4, and D6. The transcriptional silencer KAP1 binds to SETDB1, and this interaction coordinates both histone methylation and the deposition of HP1 proteins to silence gene expression; (M) The representative immunostaining images of KAP1 and SETDB1 in hTSCs and STBs (D6); (N) Western blot analysis of global H3K9me3, H3K4me3, and H3K27me3 levels in hTSCs and STB cells of D2, D4, D6; (O) The representative immunostaining images of H3K9me3, H3K4me3, and H3K27me3 in hTSCs and STBs (D6). Data are mean ± SEM. The *t*‐test is used in statistical analysis. Ns, *p* > 0.05; *p ≤ 0.05, **p ≤ 0.01; ***p ≤ 0.001; ****p ≤ 0.0001. All experiments have been independently repeated three times. Scale bars, 10 μm.

To delineate detailed senescence characteristics during hTSC‐STB development and evaluate its application potential, we investigated its aging‐related signals in multiple aspects. First, senescent cells involve impaired DNA repair and unstable genomes (Schumacher et al. 2021). In the hTSC‐STB development, a general decline in the scores of DNA repair pathways, including NER, BER, HR, NHEJ, and Fanc, at both transcriptomic (Figure 3A) and proteomic levels (Figure S3A), was evident, in line with the in vivo data (Figure S1A). Consequently, STB cells exhibited more DNA damage, as evidenced by elevated γH2AX and p53‐binding protein 1 (53BP1, marker of DNA double‐strand breaks (DSBs)) foci (Figure 3B,C). Furthermore, STB cells showed higher levels of phospho‐ATM (pATM) and reduced levels of key HR DNA repair regulator breast cancer gene 1 (BRCA1) (Figure S3B–E). To further assess DNA repair capacity, we treated hTSCs and hSTBs with etoposide (Eto) to induce DNA damage, followed by a 24 h recovery. Results showed hTSCs repaired DNA damage (low 53BP1 post‐recovery), whereas hSTBs failed to recover efficiently (Figure 3D,E). We also stained BRCA1 in this experiment, BRCA1 levels remained consistently higher in hTSCs than STBs, but were unaffected by Eto treatment or recovery in either cell type (Figure 3E, quantification not shown here). We concluded that the hTSC‐STB system is accompanied by defective DNA repair capacity and accumulated DNA damage.

The loss of nuclear scaffold proteins lamin A and lamin B has been identified as molecular markers of aging in progeria syndrome, skin decrepitude, and hippocampal degeneration (Gil 2023; Wang et al. 2017; Bin Imtiaz et al. 2021). The in vitro STBs exhibited low expression of lamin A/C (LMNA, LMNC) and lamin B1 (LMNB1) (Figure 3F–H), indicating an unstable nuclear skeleton and increased susceptibility of the genome to stress. Lamins play an important role in chromatin remodeling by interacting with the transcriptional repressor HP1 (Fischle et al. 2005). Altered HP1 distribution on chromosomes occurs in aged *Drosophila* and premature human aged cells (Wood et al. 2010; Zhang et al. 2015), whereas HP1 overexpression extends lifespan (Larson et al. 2012). The hTSCs to STBs system exhibited a clear decline of HP1α (CBX5) and HP1γ (CBX3) (Figure 3I–K). HP1 functions as part of the KRAB‐KAP1‐SETDB1 complex in the maintenance of constitutive heterochromatin (Schultz et al. 2002; Kalousi et al. 2015). Expression of the components of this repression complex was also decreased in STBs versus hTSCs (Figure 3L,M).

Emerging research emphasizes the role of epigenetic shifts, including epigenetic clocks (DNA methylation patterns that predict biological age) (Horvath 2013; Wu et al. 2024; Pal and Tyler 2016). In the development of hTSCs to STBs, we noticed a gradual decline in the repressive histone marks (H3K9me3, H3K27me3) and a rise in the active mark H3K4me3 (Figures 3N,O and S3F, accession GSE243059), coinciding with decreased lamins and heterochromatin loss seen in aged or progeria cells (Zhang et al. 2015; Wu et al. 2024; Pal and Tyler 2016; Lee, Salamah, et al. 2024). Transcriptomic analysis of histone‐modifying enzymes (writers/erasers) (Hyun et al. 2017) revealed coordinated changes aligning with these shifts in the hTSC‐STB system (Figure S3G–I). Specifically, H3K9me3 writers decreased globally (no eraser change; Figure S3G). H3K27me3 writers declined, while erasers rose significantly (Figure S3I). H3K4me3 writers and erasers showed the inverse trends (Figure S3H), consistent with the observed histone mark dynamics. Canonical histone levels, which are reported to decline with aging across species (Yi and Kim 2020; Hu et al. 2014), were significantly lower in STBs than hTSCs (mass spectrometry PXD071811; Figure S3J).

Global DNA methylation typically decreases with age in organs and tissues (Horvath 2013; Horvath and Raj 2018; Seale et al. 2022). So we checked regulators of DNA methylation, including methyltransferases and demethylases in the trophoblast system. A reduction in the expression of DNA methyltransferases DNMT1 and DNMT3B was observed in STBs (Figure S3K,L), consistent with the reported age‐related changes in MSCs and skin fibroblasts (Xie et al. 2017; Lopatina et al. 2002; Zhou et al. 2020). The expression of another member, DNMT3A, moderately increased in STBs (Figure S3K), mirroring high DNMT3A in aged human blood cells (Bond et al. 2019). For DNA demethylases, the downregulation of TET1/2/3 (10–11 translocation family) in STB cells (Figure S3M) mirrors patterns seen in premature reproductive aging (Huang et al. 2020; Unnikrishnan et al. 2018). Additionally, the thymine DNA glycosylase (TDG), which removes TET‐mediated DNA oxidation products, was also reduced (Figure S3M). These observations suggest a shared epigenetic mechanism in the hTSC‐STB system and other tissue or cell aging.

mRNA surveillance is also implicated in senescence (Kwon et al. 2023; Harries 2023). A marked mRNA surveillance pathway suppression was seen in the hTSC‐STB system (Figure S3N). N6‐methyladenosine (m6A), the predominant mammalian mRNA modification, is regulated by a core catalytic complex (including methyltransferases like 3 and 14: METTL3, METTL14) linked to aging (Liu et al. 2021). In the trophoblast model, METTL3, METTL14, and m6A complex components (RBM15, WTAP, ZC3H13) were downregulated (Figure S3O,P). Pharmacological METTL3 inhibition (STM2457 treatment) (Yankova et al. 2021) in hTSCs induced STB features with upregulated markers (CGA, CGB3, ERVW1) and concomitant suppression of proliferation‐associated genes (MKI67, PCNA) (Figure S3Q). These findings indicate that m6A methylation safeguards hTSC stemness, restraining premature differentiation and senescence.

### 2.4 High Levels of Transposable Element (TE) Expression in STBs

The human genome harbors abundant transposable elements (TEs), enriched in constitutive heterochromatin and epigenetically silenced in healthy cells (Slotkin and Martienssen 2007). Transcriptional silencing collapses (e.g., lamin depletion, heterochromatin erosion) enable TEs derepression, triggering interferon‐driven inflammation, which drives cellular senescence and tissue aging (Autio et al. 2020; Liu et al. 2023).

**FIGURE 4:**
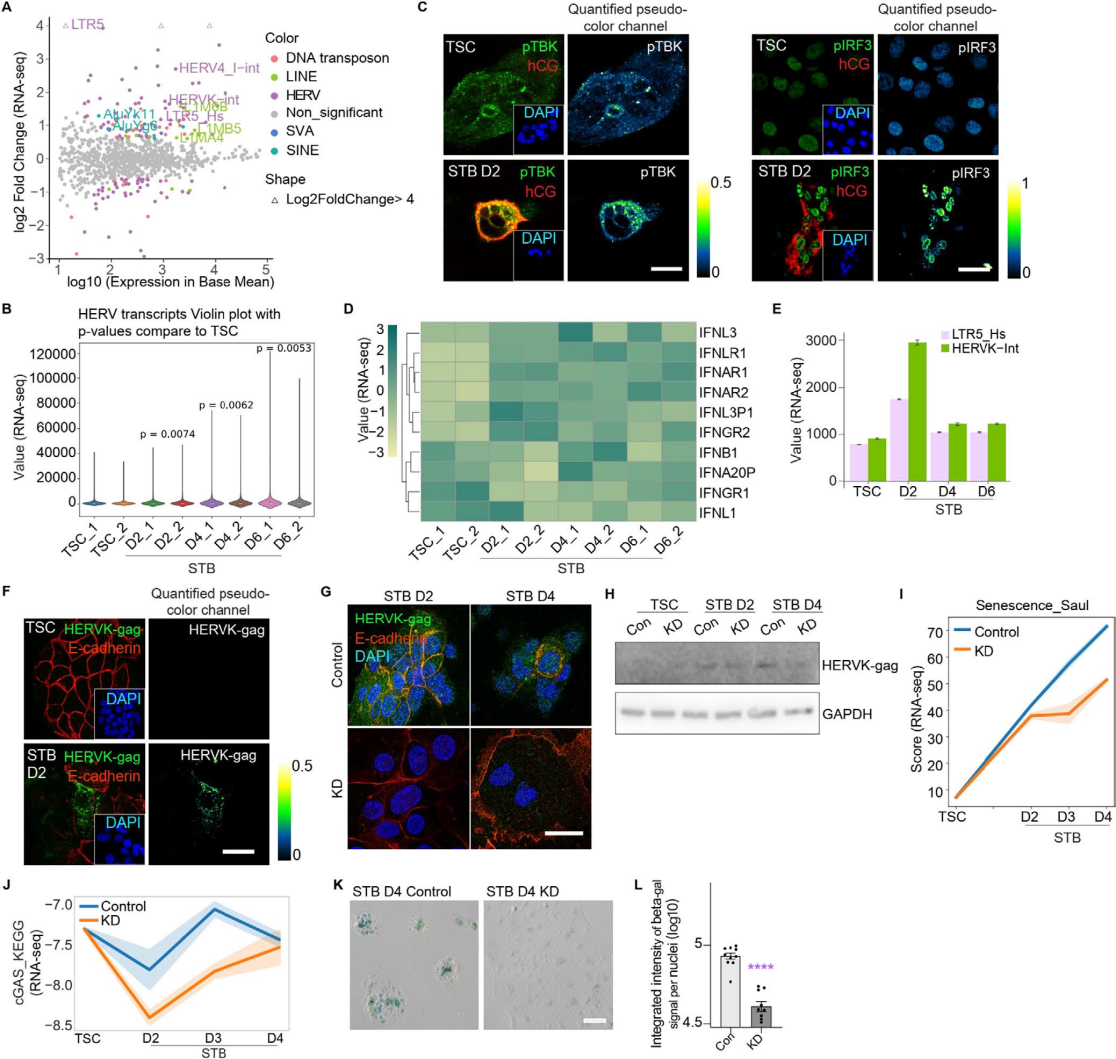
Reactivation of transposon elements (TEs) in hTSCs to STBs, which plays an important role in senescence in STBs. (A) Differentially expressed TE subfamilies in STB cells (D2) versus hTSCs. Significantly differentially expressed TEs are colored, representing their classes; (B) Violin plot of ERV transcripts with *p*‐values across hTSCs to STBs; (C) The representative co‐immunostaining images of cGAS‐STING pathway components pTBK and pIRF3 together with hCG in hTSCs and STBs (D2); (D) Heatmap of interferons expression levels along hTSCs to STBs; (E) Normalized count of HERVK‐int and its long terminal repeat LTR5‐Hs in hTSCs to STBs. In STB cells, HERVK‐int increases on D2 and decreases afterwards; (F) Representative immunostaining images of HERVK‐gag and E‐cadherin in hTSCs and STBs (D2); (G) Representative immunostaining images of HERVK‐gag and E‐cadherin in STBs of D2 and D4 cells differentiated from normal hTSCs and LTR5‐Hs KD hTSCs, respectively; (H) Western blot analysis of HERVK‐gag in hTSCs and STBs (D2 and D4) in normal (Con) and LTR5‐Hs KD group; (I) RNA‐seq analysis of senescence score changes along hTSCs to STBs of normal and LTR5‐Hs KD cells, respectively; (J) RNA‐seq analysis of cGAS‐STING pathway score in normal and LTR5‐Hs KD group; (K) Representative images of SA‐β‐gal staining in STBs (D4) of normal and LTR5‐Hs KD hTSCs; (L) Quantification of the integrated intensity of SA‐β‐gal in STB (D4) cells of normal control (Con) and LTR5‐Hs KD hTSCs. Data are mean ± SEM. The *t*‐test is used in statistical analysis. Ns, *p* > 0.05; *p ≤ 0.05, **p ≤ 0.01; ***p ≤ 0.001; ****p ≤ 0.0001. All experiments have been independently repeated three times. Scale bars, 10 μm.

During STB differentiation alongside epigenetic shifts, the expression of TEs was significantly elevated (Figures 4A and S4A). Retrotransposons are linked to cellular senescence and are detectable in aged human serum (Autio et al. 2020; Liu et al. 2023). STBs expressed considerably higher levels of key retrotransposon HERVs (Figure 4B). This coincided with the heightened cGAS‐STING pathway score (Figure S4B), increased expression of its components pTBK and pIRF3 (Figure 4C), and the downstream interferon‐related molecules (Figure 4D).

HERVK (HML‐2) (Figure S4C), the youngest integrations in humans, are highly expressed in aged cells (Autio et al. 2020; Liu et al. 2023). STB cells (notably STB D2) showed robust RNA‐level expression of HERVK elements (LTR5‐Hs, HERVK‐int; Figures 4E and S4D) and elevated HERVK‐gag protein levels (Figure 4F). Given the reported role of HERVK in aging (Liu et al. 2023), we targeted LTR5‐Hs in hTSCs using an inducible dCas9‐KRAB system (Zhang et al. 2022; Fuentes et al. 2018) (LTR5‐Hs KD), which suppressed the HERVK transcripts (gag, pol, env) and protein (HERVK‐gag) (Figures 4G,H and S4D). LTR5‐Hs KD impaired STB differentiation: STBs displayed fewer villi, reduced fusion index (Figure S4E,F), and lower expression of maturation markers (CGA, CGB, ERVW1) (Figure S4G,H).

We subsequently measured the total senescence score as in Figure 2E. The LTR5‐Hs KD STBs displayed a moderately reduced senescence score (Figure 4I), with suppressed cGAS‐STING activation (Figure 4J) and downregulated interferon signaling (Figure S4I). SA‐β‐gal staining confirmed the less senescent LTR5‐Hs KD STBs (Figure 4K,L). The senescence‐associated genes in the LTR5‐Hs KD STBs exhibited three expression patterns: downregulation (e.g., *mTOR*, *CDKN2B*, *Bcl2*), no change (e.g., *TERT*, *CDKN1A*), or upregulation (e.g., *PI3K*) (Figure S4J). However, γH2AX levels remained unchanged (Figure S4K), indicating incomplete senescence suppression with HERVK knockdown. This aligns with other studies showing HERVK repression mitigates senescence or aging to a limited extent (Autio et al. 2020; Liu et al. 2023; Hurme and Pawelec 2021), underscoring its role as a partial driver in the hTSC‐STB senescence process.

### 2.5 STBs In Vitro Exhibit Organelle Dysfunction and Senescence‐Associated Secretory Phenotypes (SASPs)

**FIGURE 5:**
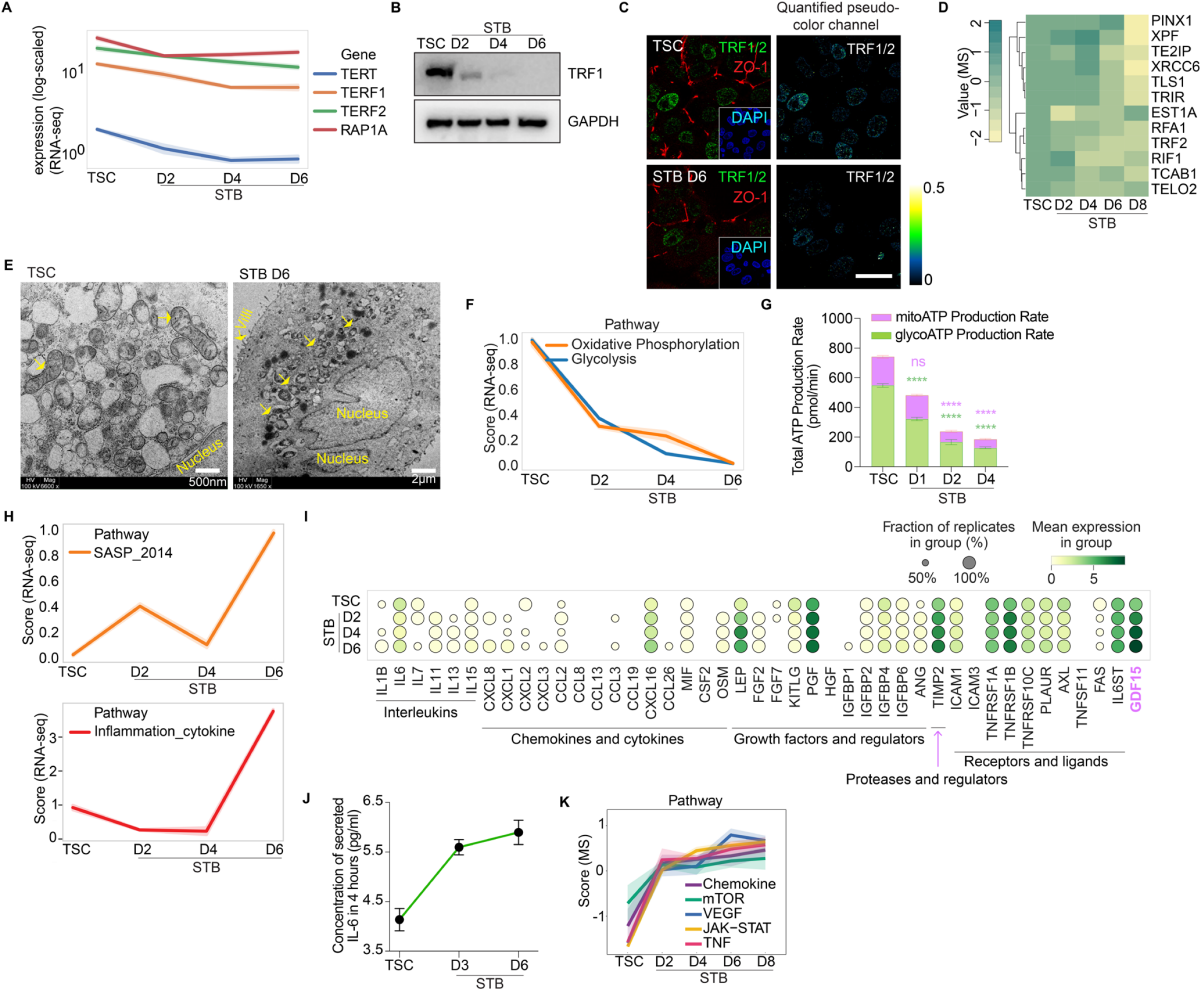
STBs display organelles dysfunction and have senescence‐associated secretory phenotypes (SASPs). (A) RNA‐seq analysis of TERT, TERF1 (TRF1), TERF2 (TRF2), and RAP1A in hTSCs and STBs; (B) Western blot analysis showing gradually lower levels of telomere capping protein TRF1 along hTSCs to STBs; (C) Representative immunostaining images of TRF1/2 in hTSCs and STBs; (D) Telomere function regulators decrease in STBs compared to hTSCs in mass spectrometry data indicating unstable and dysfunctional telomeres; (E) Representative electronic microscope images of mitochondria in hTSCs and STBs. hTSCs contain normal mitochondria with well‐defined cristae, while mitochondria in STBs are replaced with fewer cristae membrane swirls and/or voids in the center; yellow arrows point to representative mitochondria in hTSCs and STBs (D6); (F) RNA‐seq analysis of scores of oxidative phosphorylation and glycolysis pathways in hTSCs and STBs (D2, D4, D6); (G) Significantly reduced ATP production rates of oxidative phosphorylation (mitoATP) and glycosis (glycoATP) in STBs indicating mitochondria dysfunction comparing to hTSCs; (H) Line chart showing scores of pathways, SASP and sterile inflammation_cytokines, across different time points from hTSCs to STBs (D2, D4, D6) in vitro; (I) Dotplot of transcriptomics of all the well‐known SASP component genes (Interleukins, Chemokines and cytokines, Growth factors and regulators, Proteases and regulators, Receptors and ligands); (J) ELISA quantitation of IL‐6 in hTSCs and STBs (D3, D6); (K) Mass spectrometry analysis of SASP‐related pathway components (Chemokine, mTOR, VEGF, JAK–STAT, TNF) along hTSCs to STBs (D2, D4, D6). All experiments have been independently repeated three times. Two‐way ANOVA is used in statistical analysis. Ns, *p* > 0.05; *p ≤ 0.05, **p ≤ 0.01; ***p ≤ 0.001; ****p ≤ 0.0001. Scale bars, 10 μm.

Young, healthy cells maintain dynamic networks of organelles (e.g., mitochondria, telomeres) that degrade with aging (Sahin and Depinho 2010; Heiby and Ori 2022). In particular, telomere attrition drives genome instability, impairs cellular health, and directly contributes to aging and age‐linked diseases (Lopez‐Otin et al. 2023; Rossiello et al. 2022). However, telomere length did not differ between hTSCs and STBs (Figure S5A), likely because differentiation time and cell divisions in the system were limited (Figure S2E), preventing measurable telomere shortening. We thus analyzed TERT (telomerase) and shelterin complex components (TRF1, TRF2, POT1, TIN2, TPP1, RAP1), all of which showed gradual downregulation in STBs compared to hTSCs (Figure 5A–D). Telomere damage in STBs was further supported by γH2AX‐TRF1/2 colocalization (Figure S5B,C). Additionally, proteomics analysis revealed reduced expression of telomere regulators (TELO2, TE2IP, TCAB1, etc.) in STBs (Figure 5D). These results demonstrate a progressive failure of telomere maintenance mechanisms during hTSC development into STBs.

Senescent cells exhibit abnormal mitochondria biogenesis and increased mass (Gorgoulis et al. 2019; Palikaras et al. 2015; Lopez‐Lluch et al. 2008). STBs showed an elevated mitochondrial mass (MitoTracker staining, Figure S5D) and structural defects, including fragmented cristae and hollow centers, via electron microscopy (Figure 5E), mirroring mitochondrial ultrastructure in other aging models (Brandt et al. 2017). Besides morphology changes, STBs also displayed impaired respiratory function, with reduced oxidative phosphorylation (OXPHOS) and glycolysis activity (Figure 5F) and decreased ATP production from both pathways (Figure 5G), highlighting the mitochondria dysfunction from hTSCs to STBs. Senescent cells are known to accumulate reactive oxygen species (ROS) (Gorgoulis et al. 2019; Sahin and Depinho 2010; Sreedhar et al. 2020). While cytoplasmic ROS levels were similar between hTSCs and STBs, nuclear ROS intensity was markedly higher in STBs (Figure S5E). This nuclear‐specific increase likely reflects elevated DNA damage (Figure 3B,C) and diminished antioxidant capacity in the nucleus, as reported (Yakes and Van Houten 1997; Paardekooper et al. 2019; Murphy et al. 2022).

Senescent cells secrete inflammatory factors (SASPs), including cytokines, growth factors, and proteases. While STBs naturally produce pregnancy‐related factors, they showed higher SASP and inflammation scores than hTSCs (Figure 5H). RNAseq showed that most SASP components (e.g., GDF‐15) (Moon et al. 2020; Pence 2022) were upregulated in STBs (Figures 5I and S5F,G), but the classic IL‐6 (Childs et al. 2015; Tyrrell and Goldstein 2021) remained unchanged (Figure 5I). Surprisingly, several other assays (RT‐qPCR, immunostaining, ELISA) revealed elevated IL‐6 in STBs (Figures 5J and S5F,H), suggesting potential mRNA‐protein discordance in aging cells (Hipp et al. 2019). We further confirmed the enrichment of SASP‐linked pathways (mTOR, TNF, JAK–STAT, etc.) in proteomics of the hTSC‐STB system (Figure 5K). In summary, STBs display organelle dysfunction and robust SASP activation.

### 2.6 Damaged Nutrient Sensing in the hTSC‐ STB System

Most anti‐aging agents, like caloric restriction mimetics (CRMs), target the IGF/IGF1R, mTOR, and AMPK pathways (Lopez‐Otin et al. 2023; Madeo et al. 2019) (Figure S6A). In hTSC‐STB differentiation, transcriptomics revealed increased insulin resistance but reduced AMPK, IGF1, and insulin signaling (Figure S6B). Proteomics validated higher insulin resistance in STBs alongside elevated insulin secretion, AMPK, mTOR, and MAPK pathway activities (Figure S6C). Sirtuins are core nutrient‐sensing proteins that regulate aging processes and interventions (e.g., caloric restriction) and are typically reduced in aged cells (Lopez‐Otin et al. 2023; Madeo et al. 2019). While their members showed stable mRNA levels during hTSC‐STB differentiation (Figure S6D), the protein levels of SIRT1, SIRT3, and SIRT6 dropped significantly in STBs (Figure S6E–G). In contrast, the levels of SIRT2 and SIRT5, which are implicated in age‐related diseases and models (Madeo et al. 2019; Diaz‐Perdigon et al. 2020; Maxwell et al. 2011; Mao et al. 2023; Greene et al. 2019), increased in STBs (Figure S6F). The differences here in mRNA and proteomics again indicate the decoupling of mRNA‐protein in senescent cells. These shifts in nutrient‐sensing pathways align with other aging hallmarks observed in both the in vitro hTSC‐STB and the in vivo CTB‐STB systems (PI and T1) (Table S1).

### 2.7 Quantitative Evaluation of Known Anti‐Aging Molecules Using the hTSC‐STB System

**FIGURE 6:**
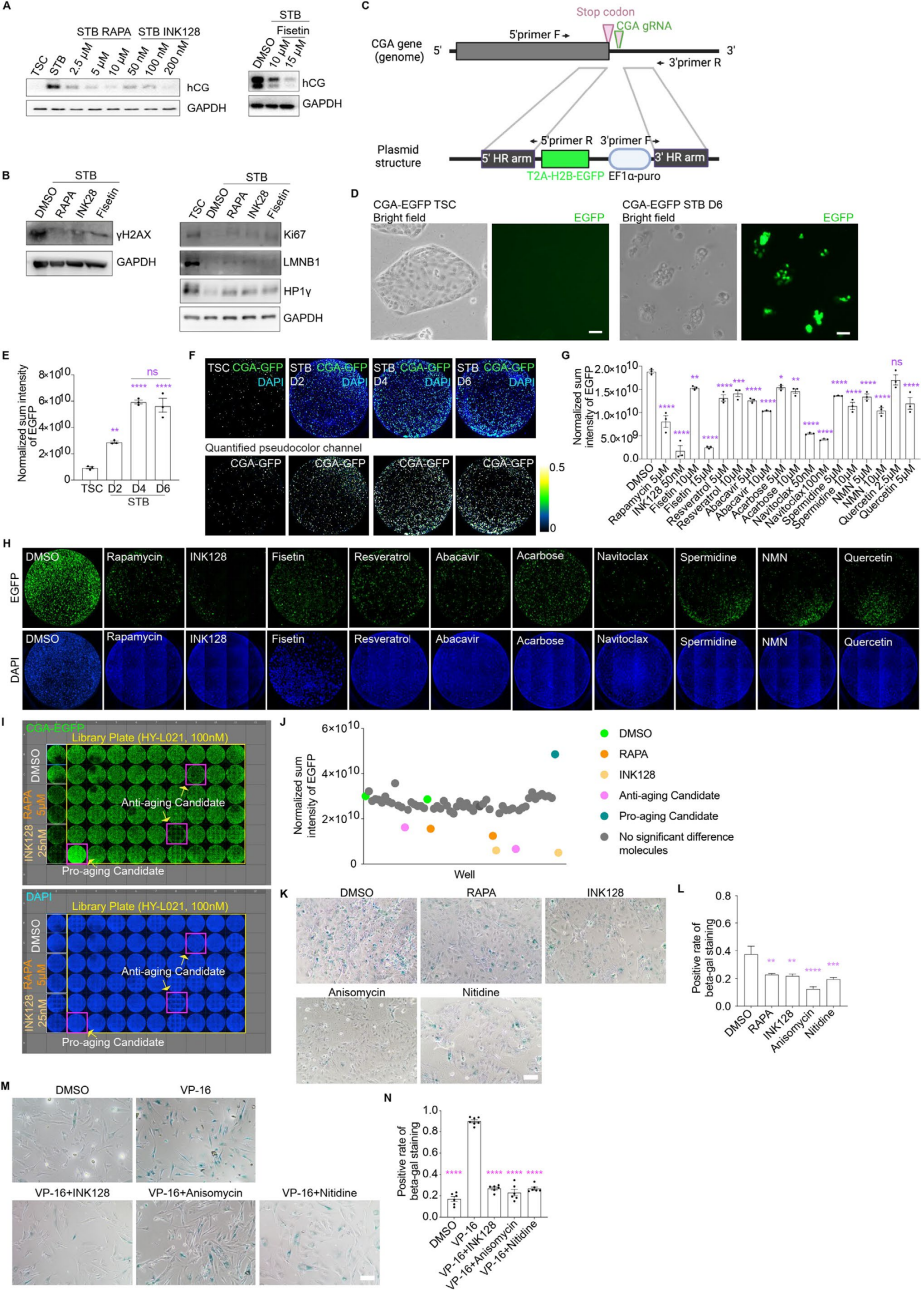
The hTSC‐STB system enables simple, fast, and quantitative evaluation of known anti‐aging molecules. (A) Western blot analysis of hCG levels in hTSCs and STBs on D2, D4, and D6 treated with different concentrations of Rapamycin (RAPA), INK128, and Fisetin. These known anti‐aging molecules are able to prevent, to some extent, hTSCs from developing into fully mature STBs; (B) Western blotting analysis showed significantly decreased γH2AX in Rapamycin, INK128, and Fisetin treated STB cells, whereas the treated STBs express higher HP1γ, Lamin B1 and Ki67 than non‐treated STBs; (C) Schematic graph of the CGA‐T2A‐H2B‐EGFP reporter allele where the T2A‐H2B‐EGFP cassette is inserted into the CGA locus for fusion peptide production and subsequent cleavage into CGA and H2B‐EGFP; (D) The CGA‐EGFP hTSC reporter cells (left) efficiently differentiate into STBs (right) with particularly high EGFP signals in the nucleus (live cells); (E) Sum intensity of EGFP gradually increases in CGA‐EGFP hTSC reporter cells to STBs. EGFP signals appear to have reached a plateau on D4 as the signals are similar in D4 and D6 cells; (F) Representative immunostaining images of CGA‐EGFP hTSCs and CGA‐EGFP STB cells of D2, D4, D6 showing EGFP signals co‐stained with DAPI; (G, H) Using the CGA‐EGFP reporter hTSCs to quantitively evaluate known anti‐aging molecules. The treatments start on day 0 of hTSCs to STBs development; Normalized sum GFP intensity refers to DAPI signals. The graphs in (G) are mean ± SEM; (I) An example of a screened 96‐well library plate. Rapamycin and INK128 were used as the positive controls. Magenta rectangle marked the potential candidates (Anti‐aging and Pro‐aging); (J) Quantification of EGFP signals of the 96‐well plate in Figure 6I. The sum intensity was normalized referring to DAPI signals; (K) β‐gal staining of STBs (D6) in the presence of candidate molecules (Anisomycin and Nitidine) in its development from hTSCs to STBs; (L) Quantification of β‐gal staining cell in (K); (M) Representative β‐gal staining images of HFF‐1 cells treated with DMSO, VP‐16, VP‐16 + INK128 25 nM, VP‐16 + Anisomycin 25 nM, and VP‐16 + Nitidine 25 nM; (N) Quantification of β‐gal staining cell in (M). One‐way ANOVA with Dunnett's test was used in statistical analysis. Ns, *p* > 0.05; *p ≤ 0.05, **p ≤ 0.01; ***p ≤ 0.001; ****p ≤ 0.0001. All experiments have been independently repeated three times. Scale bars, 10 μm.

The above findings demonstrate that the hTSC‐STB system recapitulates core aging hallmarks, positioning it as a bona fide human cellular model for aging studies with potential broad applicability. To test its utility in anti‐aging drug discovery, we evaluated three agents: mTOR inhibitors (rapamycin, INK128) and senolytic fisetin (Lopez‐Otin et al. 2023; Mannick and Lamming 2023; Yousefzadeh et al. 2018; Kaeberlein et al. 2023) (Table 2). All three compounds sharply reduced hCG levels in STBs (Figure 6A). Decreased hCG is an indicator of defective STB development. hCG produced by trophoblasts sustains pregnancy via several mechanisms, including induction of progesterone production, trophoblast fusion, vascular remodeling, and immune tolerance. Its dysregulation impacts fetal development and disease risk. Further experiments indicated that the treatments also lowered γH2AX level (Figure 6B) and modestly boosted Ki67, HP1γ, and laminB1 expressions in STBs (Figure 6B), confirming their cellular‐level anti‐aging effects.

**TABLE 2:** Information of known anti‐aging molecules.

Drug	Mechanism	Targeted hallmarks	References
Rapamycin	mTOR complex 1 (mTORC1) inhibitor	Stem cell exhaustion, Disabled macroautophagy, Mitochondria dysfunction, Cellular senescence, Loss of proteostasis, Altered intercellular communication, Chronic inflammation	Lee, Salamah, et al. (2024); Weichhart (2018)
INK128	mTORC1 and mTORC2 inhibitor	Stem cell exhaustion, Disabled macroautophagy, Mitochondria dysfunction, Cellular senescence, Loss of proteostasis, Altered intercellular communication, Chronic inflammation	Lee, Salamah, et al. (2024); Weichhart (2018)
Fisetin	Plant flavonol, Senolytics, enhancement of the antioxidant status, the inhibition of inflammation, the induction of apoptosis, etc	Altered intercellular communication, Disabled macroautophagy, Chronic inflammation, Cellular senescence	Zhou et al. (2020); Aguado et al. (2023); Justice et al. (2019)
NMN	Precursors of NAD+	Mitochondrial dysfunction, Cellular senescence, Disabled macroautophagy	Zhang et al. (2022); Yoshino, Yoshino et al. (2021)
Acarbose	Reversible inhibitor of pancreatic alpha‐amylase and membrane‐bound intestinal alpha‐glucoside hydrolase	Dysbiosis, Mitochondrial dysfunction	Wu et al. (2024); Shen et al. (2021)
Navitoclax	Active Bcl‐2 Family Protein Inhibitor	Cellular senescence, Mitochondrial dysfunction, Loss of proteostasis	Zhu et al. (2016); He et al. (2020); Robbins et al. (2021)
Resverstrol	Anti‐inflammatory effects and immunomodulating functions via SIRT1 activation	Mitochondrial dysfunction, Cellular senescence, Epigenetic alterations, chronic inflammation	Pyo et al. (2020); Gou et al. (2022)
Sperdimine	Precursor for spermine, stimulate autophagy	Stem cell exhaustion, Genomic instability, Epigenetic alterations, Mitochondrial dysfunction, Loss of proteostasis, Telomere attrition	Hofer et al. (2022)
Abacavir	Nucleoside reverse transcriptase inhibitor (NRTI), inhibit the HIV reverse transcriptase enzyme	Chronic inflammation, Genomic instability, Epigenetic alterations	Liu et al. (2023); Zhang et al. (2022)
Quercetin	Flavonoids, scavenge free radicals	Mitochondria dysfunction, Cellular senescence	Yoshida et al. (2024); Xu et al. (2018), Hickson et al. (2019), Justice et al. (2019)

To leverage the system for anti‐aging molecule screening, we proposed hCG dynamics track established aging hallmarks in this unique hTSC‐STB system to some extent. CGA combines with CGB to form intact hCG. Without CGA, functional hCG cannot be secreted, impairing placental signaling. CGA may independently regulate hTSC proliferation/differentiation (via LH/CG receptor interactions triggering cyclic AMP (cAMP)‐dependent signaling cascades) and immune tolerance at the maternal‐fetal interface (Riccetti et al. 2017). We established the component CGA expression as a marker for rapid assessment of aging activity in the hTSC‐STB system. We engineered a reporter hTSC line by inserting an EGFP cassette into the CGA locus (Figure 6C). The marked difference in CGA levels between hTSCs and STBs enabled sensitive, quantitative detection of anti‐aging effects. Tagged CGA allows real‐time tracking of differentiation efficiency and drug effects on syncytialization. We observed a progressive increase in EGFP intensity during hTSC‐to‐STB with no difference at D4 and D6 (Figure 6D–F). Treatment with known anti‐aging molecules reduced EGFP signals in the hTSC‐to‐STB transition (Figure 6G,H, Table 2), indicating detection of potential anti‐aging activities. We subsequently screened a natural product library (MedChemExpress HY‐L021; 2869 compounds) with rapamycin and INK128 as the anti‐aging positive controls (Figures 6I,J and S6H,I). Reporter hTSCs were differentiated into STBs (Day 6) in the presence of the compounds, and EGFP signals (normalized to DAPI) were quantified. Most library compounds did not have a detectable effect on STB development (strong EGFP signals), while a subset significantly reduced EGFP intensity, including known anti‐aging agents and novel candidates (Figures 6I,J and S6H,I). Interestingly, the reporter system also identified putative pro‐aging candidates (Figure 6I,J).

Among the molecules identified in the screen, anisomycin is known to alleviate neuroinflammation and Aβ/tau pathology in Alzheimer's models (Jiao et al. 2024), another candidate, nitidine, which scavenges ROS, alleviates inflammation/senescence and inhibits PI3K/Akt/mTOR signaling to induce apoptosis (Lin et al. 2022; Zhang et al. 2024). The two selected candidates were assessed for reducing β‐gal activity in STBs (Figure 6K,L). Similar experiments were performed on the HFF‐1 skin fibroblasts, where Eto (VP‐16) was used to induce cell cycle arrest. INK128, anisomycin, and nitidine all attenuated β‐gal signals in the induced aged fibroblasts (Figure 6M,N). This proof‐of‐principle screen demonstrates the hTSC‐STB system's utility for the high‐throughput discovery of novel compounds for aging interventions.

## 3 Discussion and Perspectives

Despite progress in aging research, traditional models struggle to fully replicate human aging. Knowledge derived from non‐human systems or abnormal human cells often lacks direct clinical translation. Emerging novel models such as human iPSCs, AI, and synthetic biology are transforming aging research (Pitrez et al. 2024; Nature Aging 2023; Kalyakulina et al. 2024). Patient‐derived iPSCs, for example, from progeria patients with LMNA mutations, enable an in‐depth study of aging in human genetics to test drugs like lonafarnib (Abutaleb et al. 2023). Stem cell‐derived organoids mimic tissue‐specific aging phenotypes, such as neuronal loss or metabolic decline (Pitrez et al. 2024). Emerging AI and computational tools help decode multi‐omics data to pinpoint biomarkers/drug targets. However, these models remain limited by narrow phenotypic focus, lengthy protocols, reliance on artificial stressors, data dependency, biological reductionism, etc. (Pitrez et al. 2024; *Nature Aging* 2023; Kalyakulina et al. 2024).

Aging research urgently needs human‐relevant models to study the complexity across tissues. Here, we raised up a 2D human cellular aging model, hTSC‐STB system, providing an optimized solution for aging research and drug development. Existing 2D aging models offer scalable platforms to study senescence biomarkers (e.g., p16, SA‐β‐gal) and mechanisms, while varying in induction time, key readouts, and applications. Replicative senescence (weeks–months) best mimics natural aging but is slow (Smith and Pereira‐Smith 1996), while stress‐induced models (1–3 days) offer rapid oxidative stress insights (Burnaevskiy et al. 2023). Oncogene‐induced senescence (5–10 days) links aging to cancer pathways but relies on artificial triggers. Persistent DNA damage (e.g., ionizing radiation, bleomycin) (24–72 h) tests genomic instability but may overlap with apoptosis (Wu et al. 2007). Pharmacological tools (24–48 h) enable high‐throughput screening but risk off‐target effects, whereas genetic models provide precision but require technical expertise (Mansfield et al. 2024). These 2D models lack tissue context and face technical challenges, fueling interest in alternatives.

Placental trophoblasts naturally exhibit aging hallmarks (cell cycle arrest, DNA repair decline, SASPs, epigenetic shifts, etc.), but systematic studies have been limited (Cox and Redman 2017; Gal et al. 2019). In this study, we examined in vivo trophoblast aging via single‐cell transcriptomics during STB formation from CTB. We then replicated this process in vitro in approximately one week using human hTSCs generated STBs. Despite shared 2D limitations of lacking tissue complexity, the hTSC‐STB model captures key aging hallmarks rapidly, and this system enables targeted aging studies. For instance, silencing LTR5‐Hs (HERVK's LTR) delayed STB maturation and aging, while inhibiting RNA m6A modification accelerated both. Unlike traditional 2D models, which trade speed for physiological relevance or require artificial triggers, the hTSC‐STB differentiation process is developed physiologically similar to in vivo CTB‐STB and links to normal MuSCs/HSCs aging patterns (Figures 1F and 2F). The model also detected known molecules' anti‐aging activity (such as rapamycin and fisetin) (Figure 6A,B,G,H). Thus, the hTSC‐STB system could uniquely assess aging interventions in a human‐specific context, evaluate their effectiveness and safety, accelerate both mechanistic discovery and therapeutic development, and poses better translation potential.

hTSCs can be established from embryonic/placental tissues and pluripotent stem cells, which are amenable to genetic editing, enabling the study of genetic factors and pathways and the discovery of new aging‐related genes. Human pluripotent stem cells also allow precise genome editing to create reporter cell lines that track aging. We developed a CGA‐GFP reporter hTSC line to monitor STB development in real time. This system quantifies how anti‐aging molecules affect overall STB development status. Though not a direct marker of specific aging processes, CGA‐GFP changes correlate strongly with known hallmarks, as shown by our anti‐aging molecule tests (Figure 6A,B,G–N) and tissue stem cell aging analyses (Figures 1F and 2F). This reporter system thus offers a standardized, scalable tool for screening potential anti‐aging candidates. However, candidates require validation in additional models before advancing to mechanistic studies or clinical use. In conclusion, the hTSC‐STB system fills a critical gap by providing a rapid, physiologically relevant platform that bridges the fundamental aging research with strategies aimed at extending healthy human lifespans.

### 3.1 Limitations of the System

While the hTSC‐STB system showcases aging hallmarks and offers a novel platform, several key limitations should be acknowledged:

1. Placenta‐Specific Features. The placenta is a transient organ with a lifespan of approximately 10 months. Although STBs display core aging characteristics, their biology may not fully mirror the aging processes occurring in tissues or organs that persist for decades, which may limit direct extrapolation.
2. Scope of Reporter. The current anti‐aging screening relies on reductions in hCG and CGA level within the STB culture system, which indicate disrupted maturation. The CGA‐GFP reporter is sensitive for primary screening and captures STB maturation, but does not encompass the full spectrum of aging hallmarks. Consequently, candidates identified through this reporter line could also include agents for hydatid mole/choriocarcinoma treatment and compounds that may pose risks during pregnancy. Incorporating additional reporters, such as those targeting sirtuins, cell cycle regulators, and lamins, could enable more comprehensive tracking of specific aging pathways and broaden the system's applicability.
3. Validation Needs. As a novel tool, anti‐aging candidates identified in this system require validation in complementary models such as organoids or humanized animals to confirm relevance beyond placental contexts. This step is essential to ensure translational potential and to establish that findings are applicable to other tissues and aging processes in humans.

## 4 Materials and Methods

### 4.1 Materials

**Table:** 

Reagent or resource	Source	Identifier
Primary antibody		
Anti‐GAPDH	Thermo Fisher Scientific	AM4300, RRID: AB_2536381
Anti‐HP1gamma	Thermo Fisher Scientific	MA3054, RRID: AB_2607959
Anti‐p27	Thermo Fisher Scientific	PA527188, RRID: AB_2544664
Anti‐LaminB1	Thermo Fisher Scientific	702972, RRID: AB_2784553
Anti‐p38MAPK	Thermo Fisher Scientific	338700, RRID: AB_2533144
Anti‐Ki67	Thermo Fisher Scientific	MA514520, RRID: AB_10979488
Anti‐p53	Thermo Fisher Scientific	MA512557, RRID: AB_10989883
Anti‐Sirt3	Thermo Fisher Scientific	MA514910, RRID: AB_10980823
Anti‐p16	Thermo Fisher Scientific	MA517054, RRID: AB_2538526
Anti‐RB	Thermo Fisher Scientific	MA532103, RRID: AB_2809396
Anti‐IL‐6	Thermo Fisher Scientific	MA5‐23698, RRID: AB_2609708
Anti‐Histone H3 (tri methyl K27)	Abcam	ab6002, RRID: AB_305237
Anti‐Histone H3 (tri methyl K4)	Abcam	ab8580, RRID: AB_306649
Anti‐Histone H3 (tri methyl K9)	Abcam	ab8898, RRID: AB_306848
Anti‐Trf1/2	Abcam	ab10579, RRID: AB_2201461
Anti‐LINE‐1 ORF1p	Abcam	ab245249, RRID: AB_2941773
Anti‐hCG beta	Abcam	ab9582, RRID: AB_296507
Anti‐Phospho‐Histone H2A.X (Ser139)	Cell Signaling Technology	9718, RRID: AB_2118009
Anti‐Phospho TBK1/NAK (Ser172)	Cell Signaling Technology	5483S, RRID: AB_10693472
Anti‐LMNA/C	Cell Signaling Technology	4777S, RRID: AB_10545756
Anti‐SIRT6	Cell Signaling Technology	12486S, RRID: AB_2636969
Anti‐SIRT1	Cell Signaling Technology	8469S, RRID: AB_10999470
Anti‐p21 Waf1/Cip1	Cell Signaling Technology	2947, RRID: AB_823586
Anti‐ZO‐1	ABclonal	A0659, RRID: AB_2757321
Anti‐pATM	ABclonal	AP0008, RRID: AB_2770926
Anti‐KAP1/TRIM28	ABclonal	A19568, RRID: AB_2862673
Anti‐SETDB1	ABclonal	A6145, RRID: AB_2766775
Anti‐E‐Cadherin	ABclonal	A20798, RRID: AB_3107194
Anti‐HP1α	ABclonal	A12577, RRID: AB_2759419
Anti‐HERVK‐gag	Austral Biologicals	HERM‐1831‐5, RRID: AB_10890594
Anti‐BRCA1	Proteintech	22,362–1‐AP, RRID: AB_2879090
Secondary antibody		
Anti‐mouse Alexa Fluor 594	Thermo Fisher Scientific	A21203, RRID: AB_2535789
Anti‐mouse Alexa Fluor 488	Thermo Fisher Scientific	A21202, RRID: AB_141607
Anti‐mouse Alexa Fluor 647	Thermo Fisher Scientific	A21239, RRID: AB_2535808
Anti‐rabbit Alexa Fluor 594	Thermo Fisher Scientific	A21207, RRID: AB_141637
Anti‐rabbit Alexa Fluor 488	Thermo Fisher Scientific	A21206, RRID: AB_2535792
Anti‐rabbit Alexa Fluor 647	Thermo Fisher Scientific	A21244, RRID: AB_2535812
Anti‐Rabbit HRP	Thermo Fisher Scientific	31460, RRID: AB_228341
Anti‐mouse HRP	Thermo Fisher Scientific	31430, RRID: AB_228307
Reagents and kits		
XAV939	MedChemExpress	HY‐15147
A419259 trihydrochloride	MedChemExpress	HY‐15764A
PD0325901	MedChemExpress	HY‐10254
Y‐27632	MedChemExpress	HY‐10071
CHIR‐99021	MedChemExpress	HY‐10182
Spermidine	MedChemExpress	HY‐B1776
Quercetin	MedChemExpress	HY‐18085
Navitoclax	MedChemExpress	HY‐10087
Acarbose	MedChemExpress	HY‐B0089
Etoposide (VP‐16)	MedChemExpress	HY‐13629
β‐Nicotinamide mononucleotide (NMN)	MedChemExpress	HY‐F0004
Metformin	MedChemExpress	HY‐B0627
INK‐128	MedChemExpress	HY‐13328
Rapamycin	MedChemExpress	HY‐10219
Fisetin	MedChemExpress	HY‐N0182
Abacavir	MedChemExpress	HY‐17423
Resveratrol	MedChemExpress	HY‐16561
SB‐431542	MedChemExpress	HY‐10431
DCFH‐DA	MedChemExpress	HY‐D0940
Natural compound library	MedChemExpress	HY‐L021
Insulin‐Transferrin‐Selenium‐Ethanolamine (ITS‐X) (100X)	Thermo Fisher Scientific	51500056
DMEM/F‐12, no glutamine	Thermo Fisher Scientific	21331020
1 Kb plus DNA ladder	Thermo Fisher Scientific	10787018
Pierce methanol‐free formaldehyde ampules	Thermo Fisher Scientific	28908
Chorionic gonadotropin monoclonal antibody (FB12)	Thermo Fisher Scientific	14‐6508‐82
Hoechst 33258	Thermo Fisher Scientific	H21491
DAPI (4′,6‐diamidino‐2‐phenylindole, dilactate)	Thermo Fisher Scientific	D3571
Geltrex LDEV‐free reduced growth factor basement membrane matrix	Thermo Fisher Scientific	A1413302
Gibco EGF recombinant human protein	Thermo Fisher Scientific	PHG0313
NUPAGE 4X LDS sample buffer	Thermo Fisher Scientific	NP0008
Pierce BCA protein assay	Thermo Fisher Scientific	23227
MitoTracker green	Thermo Fisher Scientific	M7514
Penicillin–streptomycin‐glutamine (100×)	Thermo Fisher Scientific	10378016
CTS (cell therapy systems) N‐2 supplement	Thermo Fisher Scientific	A1370701
B‐27TM supplement (50×), serum free	Thermo Fisher Scientific	17504044
Neurobasal medium	Thermo Fisher Scientific	21103049
FBS	Thermo Fisher Scientific	10270
LIF recombinant human protein	Thermo Fisher Scientific	PHC9484
PowerUp SYBR green master mix	Thermo Fisher Scientific	A25776
MicroAmp fast optical 96‐well reaction plate with barcode, 0.1 mL	Thermo Fisher Scientific	4346906
MicroAmp optical adhesive film	Thermo Fisher Scientific	4360954
Pierce dimethylsulfoxide (DMSO), LC–MS grade	Thermo Fisher Scientific	85190
Knockout serum replacement	Thermo Fisher Scientific	10828010
β‐Mercaptoethanol	Thermo Fisher Scientific	31350010
TryPLE‐Express	Thermo Fisher Scientific	12605036
Minimum essential medium (MEM) vitamin solution	Thermo Fisher Scientific	11120052
Triton X‐100	Sigma‐Aldrich	T9284
L‐Ascorbic acid	Sigma‐Aldrich	A8960‐5G
Forskolin	Sigma‐Aldrich	F3917
SpeI‐HF—500 units	New England Biolabs (NEB)	R3133S
NotI‐HF—500 units	New England Biolabs (NEB)	R3189S
SalI‐HF—2000 units	New England Biolabs (NEB)	R3138S
T4 DNA Ligase—20,000 units	New England Biolabs (NEB)	M0202S
AscI	New England Biolabs (NEB)	R0558S
BbsI‐HF	New England Biolabs (NEB)	R3539L
Human hCG ELISA kit	Abcam	ab100533
Human IL‐6 ELISA kit	ABclonal	RK00004
μ‐slide 8 well high glass bottom	ibidi	80807
Proteinase K	Takara Bio	9034
2× Phanta max master mix	Cellagen Technology	P515‐02
Senescence β‐galactosidase staining kit	Cell Signaling Technology	9860
A83‐01	Tocris	2939
TaKaRa MiniBEST plasmid purification kit Ver.4.0	BIO‐STATION LIMITED	9760
Matrigel matrix. GFR	Corning	354230
Bovine serum albumin frac V 100 mL	Life Technologies	15260037
Valproic acid (Sodium salt)	Stem cell technology	72292
Software		
ImageJ 1.47v	https://imagej.net/software/fiji/	
GraphPad Prism 9	https://www.graphpad.com/	
Excel	https://products.office.com/excel	
BioRender	https://www.biorender.com/	
Adobe Illustrator 26.4.1	https://www.adobe.com/hk_en/	

### 4.2 Methods

#### 4.2.1 Cell Culture

All cell cultures were maintained in an incubator at 37°C, 5% CO_2_ with saturating humidity and tested negative for mycoplasma contamination weekly.

Man1(M1) hEPSCs were converted from primed hESCs, and hEPSC‐em were derived from human pre‐implantation embryos (Chen et al. 2023). All hEPSC lines were cultured on pre‐seeded γ irradiation‐inactivated SNL feeder layers and passaged every 3–4 days after reaching an 80%–90% confluency.

The hEPSC medium (refer to Gao et al. 2019) is an N2B27‐based media supplemented with 5 mM XAV939, 1 mM CHIR99021, and 0.1 mM A‐419259. N2B27 basal media (Gao et al. 2019) is prepared as a 1:1 of DMEM/F12 and neurobasal medium supplemented with 0.5× N2 supplement, 1× B27 supplement, 1× ITS‐X, 50.0 mM 2‐mercaptoethanol, 1% penicillin–streptomycin‐glutamine, 1× non‐essential amino acid solution, and 50 mg/mL vitamin C.

The hTSC lines were derived from M1 hEPSC and hEPSC‐em cells differentiated in hTSC medium. hTSC lines were seeded on plates pre‐coated with 1% Matrigel/Geltrex, cultured in hTSC medium, and passaged every 4–5 days at a 1:4–1:6 ratio. hTSCs differentiated into STBs via rinsing the cells into STB medium for 5–6 days. hTSC medium was prepared as DMEM/F12 supplemented with 0.1 mM 2‐mercaptoethanol, 0.2% FBS, 0.5% penicillin–streptomycin, 0.3% BSA, 1% ITS‐X supplement, 1.5 μg/mL L‐ascorbic acid, 50 ng/mL EGF, 2 μM CHIR99021, 0.5 μM A83‐01, 1 μM SB431542, 0.8 μM VPA and 5 μM Y27632. STB medium was prepared as DMEM/F12 supplemented with 0.1 mM 2‐mercaptoethanol, 0.5% penicillin–streptomycin, 0.3% BSA, 1% ITS‐X supplement, 2.5 μM Y27632, 2 μM forskolin, and 4% knock‐out serum (KSR).

The inducible LTR5‐Hs knockdown (KD) hESC line, cultured in mTESR1, was a gift from Professor Wei Jiang at Wuhan University. We first cultured and subcloned the LTR5‐Hs KD hESCs in hEPSC medium for several passages to get stable hEPSC lines, and then hTSC lines were derived from these hEPSCs as described before (Gao et al. 2019). To efficiently achieve LTR5‐Hs KD, 1 μg/mL doxorubicin (DOX) was added to the hTSCs culture for more than one week.

Human foreskin fibroblasts (HFF‐1) were obtained from the University of Hong Kong—Shenzhen Hospital (Shenzhen, China) and cultured in M15. M15 includes DMEM, 15% FBS, and 1% penicillin–streptomycin. The medium was changed every 2 days, and cells were passaged or used to perform experiments when the confluency reached 80%–90%.

### 4.3 Senescence β‐Galactosidase Staining

Samples of hTSCs and STBs were fixed with 1 mL of 1X Fixative Solution (Cell Signaling Technology (CST), #9860) to each 35 mm well, for 10–15 min at room temperature (RT). All the subsequent steps precisely referred to the instructions of the vendor (CST, #9860). Samples were checked under Nikon Eclipse Ti2 Inverted Microscope Systems, and images were further processed and analyzed with ImageJ 1.47v.

### 4.4 Enzyme‐Linked Immunosorbent Assay (ELISA)

Fresh hTSC and STB medium were added to hTSCs and STBs at around 60%–70% confluency. The cultured medium of hTSCs and STBs was collected after 4 h. After centrifuging, the cultured medium, together with sterile non‐cultured hTSC and STB medium, was used to perform ELISA tests, referring to the instructions of the Human IL‐6 ELISA Kit (ABclonal, RK00004) and the Human hCG ELISA Kit (Abcam, ab100533). Data were read with a plate reader and processed under Prism 9 (GraphPad).

### 4.5 CGA‐T2A‐H2B‐EGFP hTSC Reporter Cell Line Generation

Based on the template plasmid pCD31‐5HA‐T2A‐H2B‐EGFP‐loxp‐EF1a‐puro‐loxp‐3HA and pSpCas9(BB)‐2A‐Puro (PX459) V2 (Addgene: #62988) in our lab, we picked the gene CGA for reporter cell line system generation due to the huge difference in CGA expression between hTSCs and STBs. First, we got the full sequences of the human CGA gene from the website *Ensembl genome browser 110*. We designed the gRNAs for the gene right after the stop codon (less than 100 bp away from the stop codon) and inserted the gRNAs into the pSpCas9(BB)‐2A‐Puro (PX459) V2 construct by digestion and T4 ligation method. Then we got around 1000 bp homologous arms, both 5′HA and 3′HA parts, right in front of the stop codon and right after the gRNA‐targeted site, respectively. With the homologous recombination method in the ClonExpress II One Step Cloning Kit, we inserted both 5′HA and 3′HA parts of each gene into the pCD31‐5HA‐T2A‐H2B‐EGFP‐loxp‐EF1a‐puro‐loxp‐3HA to get the CGA‐T2A‐H2B‐EGFP reporter constructs. After that, we transfected the gene reporter plasmid together with the paired gRNA plasmid into the hEPSC lines. With puromycin selection for 7 days, we picked the hEPSC colonies and confirmed through genotyping for the reporter insertion in the genome. Then we expanded the right hEPSC colonies and differentiated to CGA‐T2A‐H2B‐EGFP hTSC reporter cell line for further study.

### 4.6 Mass Spectrometry and Proteomic Data Analysis

For the experiment, at least 1 million cells per sample of hTSC and STB (on Days 2, 4, 6, and 8) were collected. Sample processing and liquid chromatography‐mass spectrometry (LC–MS/MS) analysis were performed at LKS Faculty of Medicine, Proteomics and Metabolomics Core Facility (PMcore), Centre for PanorOmic Sciences (CPOS), the University of Hong Kong. Raw mass spectrometry data were processed using Spectronaut. Direct DIA data analysis was performed on Spectronaut v.14 using default settings without N‐acetyl variable modification enabled. Trypsin specificity was set to two missed cleavages and a protein and PSM false discovery rate of 1%, respectively. Data filtering was set to Q‐value, and normalization was set to global normalization. Results were then exported to an Excel file (Microsoft) for further processing. For proteome quantification, the Excel file was processed in R Studio v4.2.2. Excel files with normalized MS data were read in. Average log2 ratios comparing to the control (baseline) were normalized and standardized protein‐wise to a range from −1 to 1 using Z‐score and rescale function built in the “scale” R Package across samples. Proteins of MS with any missing data were excluded from further visualization. Protein expression levels along time were illustrated using the “heatmap.2” function from the “gplots” R Package, with the baseline treated as 0.

### 4.7 ChIP‐Seq Analysis

The raw data were trimmed by Trim galore (v0.6.10) (Krueger and Galore 2015) with default parameters. Trimmed reads were mapped to the human reference genome (GRCh38) using Bowtie 2 (v2.5.2) (Langmead and Salzberg 2012) with default parameters. SAMtools (v1.18) (Li et al. 2009) was used to retain the paired and unique mapped reads and to remove PCR duplicated reads. bamCoverage provided by deepTools (v3.5.2) (Ramirez et al. 2016) was employed with parameters “‐bs 200‐normalized using RPKM” to produce the BW file. MACS3 (v 3.0.0b3) (Zhang et al. 2008) was used for peak calling with parameters “‐f BAMPE ‐g hs—nomodel ‐q 0.05”, with an additional parameter “—broad” for H3K9me3. For the profile plot, peaks of the same histone modification from all samples were merged using BEDTools (v2.31.1) (Quinlan and Hall 2010). The peak summits were further refined by MAC3. plotProfile provided by deepTools was then used to produce a profile plot.

### 4.8 Electroporation and Transfection

Electroporation of M1 hEPSC and hEPSC‐em were performed with the Invitrogen NeonTransfection System. hEPSCs were dissociated into single cells with TrypLE or 0.05% Trypsin–EDTA. M10 medium (DMEM with 10% FBS) was added to neutralize the trypsin. After centrifugation, 20–40 μL R buffer in the Neon kit was used to resuspend hEPSC cells and mixed with plasmids. The cells were then seeded with a recovery medium (EPSC medium + 10% FBS + 10 μM Y27632). After incubation overnight, cells were switched to a typical hEPSC medium. 48 h later, 1 mg/mL Puromycin was used for selection. After 5–7 days, single colonies were picked, genotyped, and expanded for further experiments.

### 4.9 Western Blotting

The proteins were extracted with RIPA lysis (Thermo Fisher Scientific, 89901) and Pierce Protease Inhibitor Mini Tablets (Thermo Fisher Scientific, A32953). Protein concentrations were measured with Pierce BCA Protein Assay Kits (Thermo Fisher Scientific, 23227). Then samples with a certain amount of NuPAGE LDS sample buffer (4×) (Thermo Fisher Scientific, NP0008) were boiled at 95°C for 10 min. Boiled samples were loaded with the same amount and separated in 12% polyacrylamide gels (Thermo Fisher Scientific, NP0343BOX) and transferred to PVDF membranes using the Bio‐Rad transblot turbo system according to the manufacturer's guidance. Images were developed by the ChemiDoc Imaging System and processed with ImageJ 1.47v. Primary antibodies were used at a dilution ratio of 1:1000, while secondary antibodies were incubated at a dilution ratio of 1:2000.

### 4.10 Immunofluorescence Staining

Samples were seeded in a μ‐slide 8‐well dish (ibidi, 80826) pre‐coated with Matrigel/Geltrex at a density of 1–2 × 10^4^ cells per well and fixed at room temperature with 4% paraformaldehyde (PFA) for 15–30 min. Afterwards, 0.05% Triton X‐100 in PBS was utilized to permeabilize for 10–15 min, and 2% FBS in PBS was used for blocking for 1–2 h. Followed by incubation with primary antibodies in a 4°C fridge overnight. Then wash 3–5 times, 5–10 min per time on the shaker. Secondary antibodies were incubated at room temperature for 1–2 h. Another 3–5 rounds of washing in PBS were done and stained with DAPI or Hoechst for 10–15 min to mark nuclei. At last, samples were imaged under a Nikon Eclipse Ti2 NIS elements AR fluorescence microscope.

### 4.11 Mitotracker and DCFH‐DA Staining

hTSCs and STBs were seeded in a μ‐Slide 8‐well dish pre‐coated with Matrigel/Geltrex. 50 nM MitoTracker Green^FM^ (Invitrogen, M7514) and 5 μM DCFH‐DA (MedChem Express, HY‐D0940) were added to the dish, respectively. Wait for 30 min and wash the cells with sterile PBS. Then fix the cells with 4% PFA for 10–15 min at RT and stain with DAPI after permeabilization for 10–15 min with 0.05% Triton X‐100 in PBS. The slides were imaged and processed similarly to immunofluorescence staining samples.

### 4.12 DNA Repair Capacity Test

The DNA damage response test was performed as described previously (Fu et al. 2021). In brief, TSCs and day 4 STBs were exposed to 2.5 μM etoposide for 2 h to induce DNA damage. Afterwards, cells were released from the treatment after washing with PBS twice. Untreated cells (0 h), cells under exposure to etoposide for 2 h (2 h), and cells after 24 h releasing from etoposide (24 h) were collected for immunofluorescence microscopy of 53BP1 (#4937, Cell Signaling Technology, 1:300) and BRCA1 (sc‐6954, Santa Cruz, 1:300). More than 50 cells were counted for quantification of each group.

### 4.13 Reverse Transcription‐Quantitative Polymerase Chain Reaction (RT‐qPCR)

The total RNA of each sample was extracted with Trizol (Life Technology, 391,501). The concentration of the RNA samples was measured with Nanodrop, and 1 μg of each was reverse transcribed into cDNA with PrimeScript RT Master Mix (Takara: RR036A) on a thermal cycler. The PowerUpSYBR Green Master Mix was used for amplifying genes. Primer sequences are listed in Table 3. All qPCR experiments were performed on The StepOnePlusTM Real‐Time PCR System (Applied Biosystems). Gene expression levels were normalized to GAPDH and were analyzed in Prism 9 (GraphPad).

**TABLE 3:** RT‐qPCR primers.

Gene	Forward primer	Reverse primer
GAPDH	CCTCAACGACCACTTTGTCAAG	TCTTCCTCTTGTGCTCTTGCTG
IL1 alpha	TGTATGTGACTGCCCAAGATGAAG	AGAGGAGGTTGGTCTCACTACC
CGA	TCCATTCCGCTCCTGATGTGCA	CGTCTTCTTGGACCTTAGTGGAG
CGB3	ACCGTCAACACCACCATCTGTG	GAAGCGCACATCGCGGTAGTTG
ERVW1	CGCAACTGCTATCACTCTGCCA	CAGACAGTGACTCCAAGTCCTC
CSH1	CCT CCA ACA TGG AGG AAA CGCA	GTT GGC GAA CAT ACT CCT GAG G
HERVK env	GCTGCCCTGCCAAACCTGAG	CCTGAGTGACATCCCGCTTACC
HERVK gag	AAATAAGACCCAACCGCCAGTAGC	GAATTGCCATGCCTCAGTATCTCC
HERVK pol	GCCGATGAAAAAGCCCGTAAGG	TTGACACTCAGGATTGGCGTTTTC
IL8 (CXCL8)	GAGAGTGATTGAGAGTGGACCAC	CACAACCCTCTGCACCCAGTTT
Syndecan 1 (SDC1)	TCC TGG ACA GGA AAG AGG TGCT	TGT TTC GGC TCC TCC AAG GAG T
IL1 beta	CCACAGACCTTCCAGGAGAATG	GTGCAGTTCAGTGATCGTACAGG
IL‐6	AGACAGCCACTCACCTCTTCAG	TTCTGCCAGTGCCTCTTTGCTG
CCL2	AGAATCACCAGCAGCAAGTGTCC	TCCTGAACCCACTTCTGCTTGG

### 4.14 Telomere Measurement by Real‐Time qPCR

Genome DNA was prepared using the DNeasy Blood & Tissue Kit (QIAGEN, Valencia, CA). Average telomere length was measured from total genomic DNA using a real‐time PCR assay. PCR reactions were performed on the Bio‐Rad CFX Opus 96 System, using telomeric primers, primers for the reference control gene (human 36B4 single‐copy gene), and PCR settings as described (Hastings et al. 2024). For each PCR reaction, a standard curve was made by serial dilutions of known amounts of DNA. The telomere signal was normalized to the signal from the single‐copy gene to generate a T/S ratio indicative of relative telomere length. An equal amount of DNA (20 ng) was used for each reaction. 36B4 primer: 5′‐CAGCAAGTGGGAAGGTGTAATCC‐3′ and 5′‐CCCATTCTATCATCAACGGGTACAA‐3′; Tel primer: 5′‐GGTTTTTGAGGGTGAGGGTGAGGGTGAGGGTGAGGGT‐3′ and 5′‐TCCCGACTATCCCTATCCCTATCCCTATCCCTATCCCTA‐3′.

### 4.15 Metabolic Profiling by Seahorse XFe96

Human TSCs were seeded in XFe96 culture microplates with a cell density of 2 × 10^4^ two days before the metabolic measuring. Metabolic profiling of STBs was conducted by seeding 2 × 10^4^ human TSCs in XF96 culture microplates and allowed for differentiation for 4 days. On the day of the metabolic profiling, the cell culture medium was replaced by DMEM XFe Medium, pH 7.4 supplemented with 10 mM of glucose, 1 mM of pyruvate, 2 mM of glutamine, and cells were incubated for 45 min at 37°C in a non‐CO_2_ atmosphere. Then the medium was replaced with pre‐warmed DMEM XFe Medium, pH 7.4 (supplemented), and cells were subjected to metabolic measuring. The Real‐Time ATP Rate Assay Kit was used to measure the ATP production rate of human TSCs and STBs, with the following reagent concentrations: 1.5 μM for oligomycin and 0.5 μM for rotenone/antimycin A. Reagents were loaded into the sensor cartridge as indicated by the manufacturer during the metabolic measuring. The equipment was calibrated following the manufacturer's instructions. After completing the calibration, the OCR and ECAR were measured before and after the sequential injection of the reagents of the assay kit. ATP production was calculated using the Agilent Seahorse Wave Controller 2.6 software according to the manufacturer's instructions. All the data obtained was normalized to the cell number. The metabolic profiling was performed using the Agilent Seahorse XFe96 Analyzer at the Centre for PanorOmic Sciences (CPOS) at the University of Hong Kong.

### 4.16 High Content Confocal Screening

hTSCs were seeded on 1% Matrigel/Geltrex‐coated 96‐well Flat Clear Bottom Black Polystyrene TC‐treated Microplates for STB differentiation. For library screening, the library plates (MedChemExpress, HY‐L021, total 2869 compounds) were diluted to working concentration first. DMSO and molecules (library plates or well‐known anti‐aging molecules) were used to treat hTSCs to STBs according to different working concentrations. Waiting for 4–6 days for the STB maturation, plates were fixed with 4% PFA and stained with DAPI/Hoechst. Then Large images were generated and analyzed with the Nikon Spinning Disk Confocal Imaging System. Image processing and quantifications were performed using ImageJ 1.47v.

### 4.17 Electronic Microscopy

Around 300,000 cells per mL of trypsinized hTSC and STB cells were fixed with EM fixative (4% PFA or 2.5% GTA) at 4°C overnight. Then put in sharp bottom micro‐centrifuge tubes (1.7 mL) and brought in an ice bath to the Electronic Microscopy laboratory (Queen Mary Hospital) for further sample preparation. Afterwards, samples were checked with the Philips CM100 TEM system, and different magnification images were taken for further analysis.

### 4.18 Statistical Analysis

All produced datasets had performed at least three independent repeats. Datasets were imported into Prism 9 (GraphPad). Statistical graphs with the mean and SD/SEM were plotted via unpaired two‐tailed Student's *t*‐test, two‐way ANOVA, and one‐way ANOVA with Dunnett's test. No significance (ns), *p* > 0.05; *p ≤ 0.05, **p ≤ 0.01; ***p ≤ 0.001; ****p ≤ 0.0001.

### 4.19 Data Availability

scRNA‐seq dataset of peri‐implantation trophoblast from Zhou et al. is publicly available (accession GSE109555). The annotation was obtained from Castel, et al. (https://gitlab.univ‐nantes.fr/E137833T/Castel_et_al_2020/‐/tree/master). Among the 5911, 8–14 embryonic day cells, late TE stage to STB differentiation was subsetted, and AME‐like cells were identified using canonical markers and removed, ending up with 3238 cells. scRNA‐seq datasets of the first‐trimester placenta are available from Vento‐Tormo et al. (2018) (13,777 cells from Carnegie stage 14 to 10‐week post‐fertilization human stage, accession E‐MTAB‐6701) and Shannon et al. (2022) (3854 cells from 6 to 7 weeks' gestational age to 11–12 weeks gestational age, accession GSE174481), respectively. For term‐stage placenta, averaged scRNA count across CTB of 3 stages and bulk RNA‐seq of STB from Pavlicev et al. (2017) (accession GSE87720). Bulk RNA‐seq of TSC differentiation is available from Ruan et al. (2022) (accession GSE190432). The CHIP‐seq of H3K9me3, H3K27me3, and H3K4me3 is available from Lee, Salamah, et al. (2024) (https://www.ncbi.nlm.nih.gov/geo/query/acc.cgi?acc=GSE243059). The result file of mass spectrometry was available in Table S2, and all the raw data can be found on PRIDE website (https://www.ebi.ac.uk/pride/, Reviewer access: Project accession: PXD071811, Token: otfPRZ0sSICy).

### 4.20 Code Availability

Codes used to generate figures in this paper are available in the GitHub repository (https://github.com/leeyoyohku/Aging).

### 4.21 Transcriptome‐Wide Correlation Analysis

All transcriptomes are concatenated, among which single‐cell data were summed into pseudobulk according to the specific group (stage × cell type) using ADPBulk. The Pearson correlation coefficients were computed between each pair of groups based on all overlapping genes. The cluster maps were then plotted using seaborn, with linkage being computed using the “weighted” method.

### 4.22 Aging Signature Scores

Each transcriptome is normalized to millions of counts per cell for downstream processing. Gene lists for aging‐related pathways were obtained through KEGG, REACTOME, and selected manuscripts (See Table S1 for gene lists). The weighted average score of each pathway was then computed for each cell/sample in the transcriptome. More details are available in the Scanpy documentation (scanpy.tl.score_genes: https://scanpy.readthedocs.io/en/latest/generated/scanpy.tl.score_genes.html).

### 4.23 Cross‐Comparison With MuSC or HSC Aging

1902 TNFRSF12A+ 15+ years old MuSCs from Muscle Aging Cell Atlas (Kedlian et al. 2024) and 3568 15+ years old HSCs from Bone Marrow Atlas (Lee, Hodzic Kuerec, and Maier 2024) were preprocessed, including normalized and log‐transformed, before computing 50 principal components (PCs). A linear regression model was fitted to the 50 PCs of the two‐reference data with the cell donor's chronological age as the response variable. Integration of trophoblast datasets with the MuSC or HSC dataset was performed using ingest (sc.tl.ingest). For MuSC data, we used the mean age from each age range (categorical). The linear regression model could be used to predict the age of each cell from trophoblast datasets using the integrated PC values. 25% of each dataset was used for self‐predictions.

### 4.24 GO and GSEA Analyses

Differentially methylated genes were identified using the Mann Whitney test and filtered with a *p*‐value cutoff = 0.1 and log fold change > 1. Gene enrichment and ontology analyses were then performed on these differential genes using the gseapy package. Top items with significant enrichment *p*‐values were visualized using dot plots.

### 4.25 Analysis of Transposable Elements

Transposable element annotations were obtained from the UCSC Genome Browser using RepeatMasker. SQuIRE was utilized in ‘total’ mode to quantify TE expression. Differential expression of TEs was analyzed using DESeq2. TEs with an expression fold change greater than 1.5, and an adjusted *p*‐value less than 0.05 were considered significantly differentially expressed.

## Author Contributions

P.L. conceived the concept and edited the manuscript. Z.F. performed most experiments. C.S.L., W.Z., and Y.H. analyzed genomics data. H.F. performed the telomere‐related experiments, established the LTR5‐Hs KD hTSC line, and conducted LTR5‐Hs KD‐related STBs differentiation experiments. Y.H. performed the Seahorse experiments. W.J. analyzed proteomics data. Z.F. and C.S.L. wrote and edited the manuscript. All authors had proofread the manuscript.

## Funding

This work in P.L.'s lab at the HKU and Centre for Translational Stem Cell Biology was financially supported by the InnoHK initiative of the Innovation and Technology Commission of the Hong Kong Special Administration Region Government; GRF (RGC No. 17109924); National Science Foundation of China; and RGC (NSFC/RGC Collaborative Research Scheme CRS_HKU703).

## Conflicts of Interest

The authors declare no conflicts of interest.

## Supporting information


**Data S1:** acel70352‐sup‐0001‐FigureS1‐S6.pdf.

**Table S1:** Gene lists for aging‐related pathways in transcriptome analysis of hTSCs and STBs.

**Table S2:** Direct Data‐Independent Acquisition (DIA) analysis results of mass spectrometry data in the hTSC‐STB differentiation process.

## Data Availability

The data that support the findings of this study are available on request from the corresponding author. The data are not publicly available due to privacy or ethical restrictions.
