# Supplementary material for: A Novel Human Cellular System for Studying Normal Aging and for Anti‐Aging Discovery
Source: Aging Cell. 2026 Jan 20;25(2):e70352. doi: 10.1111/acel.70352 (PMC12816977; doi:10.1111/acel.70352)
Supplement: Supplementary file 1 — Data S1: acel70352‐sup‐0001‐FigureS1‐S6.pdf. [file ACEL-25-e70352-s001.pdf]

Supplementary Materials for

A Novel Human Cellular System for Studying Normal Aging and for Anti-Aging Discovery

Zhen Feng, Cheuk Shuen Li, Haifeng Fu *et al.*

Corresponding author: Pentao Liu, Ph.D. E-mail: pliu88@hku.hk

Supplementary Figures

Figure S1

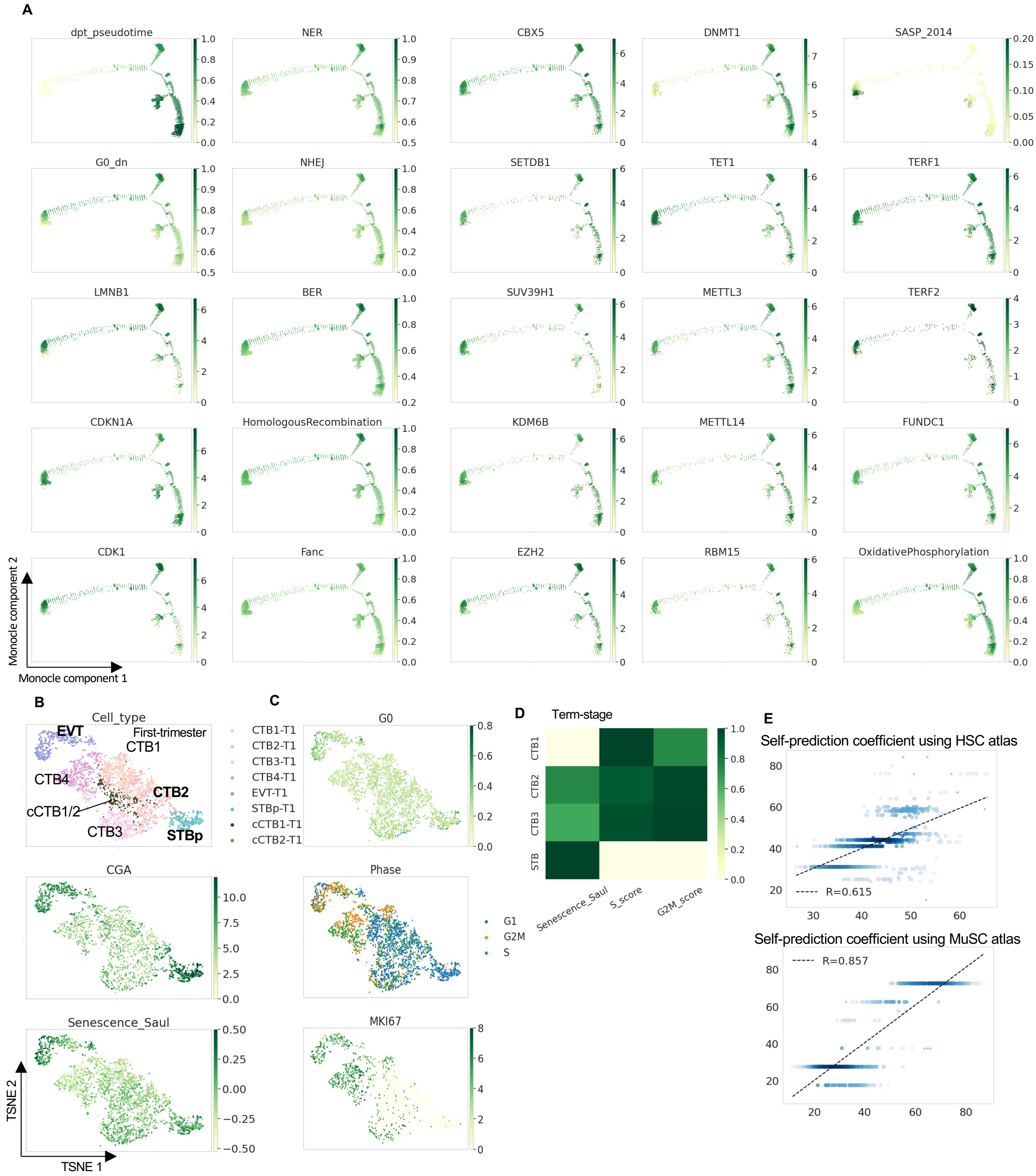

Figure S1. Placenta syncytiotrophoblast development exhibits senescence hallmarks. **A.** Embeddings same as Fig. 1D. Colors suggest pathways indicative of different aging hallmarks, including cell cycle arrest, genome instability, heterochromatin and epigenetic aberrations, intercellular alterations, telomere and mitochondrial dysfunctions, from upper left to lower right, respectively; **B.** Tsne plots displaying the differentiation and overall senescence trend along the differentiation trajectory from CTB to EVT (upper left) and syncytiotrophoblast

precursors (STBp, lower right) during the first-trimester stage (3854 cells, Shannon, 2022). CTB2 was identified as the progenitor for other CTB for other CTB subclusters as well as STBp). cCTB: column CTB; **C.** Embeddings same as B. Colors suggest a shift in the cell cycle phases, including an increase G0 level (upper), a switch from G2M/S-phase to G1 (middle), and proliferation marker Ki67 (MKI67); **D.** Heatmap showing scaled scores for G2M and S cell phase as well as senescence pathway in term-stage placenta (Pavlicev, 2017). CTB1-3 are trophoblast cells of different stages specified in the original paper. Values are normalized and scaled from 0-1; **E.** Self-prediction scores of the linear regression models used in Fig 1F. The X-axis indicates the predicted donor ages for the test set, while the y-axis indicates the real donor ages. Linear regression coefficients were labelled in the top left corner.

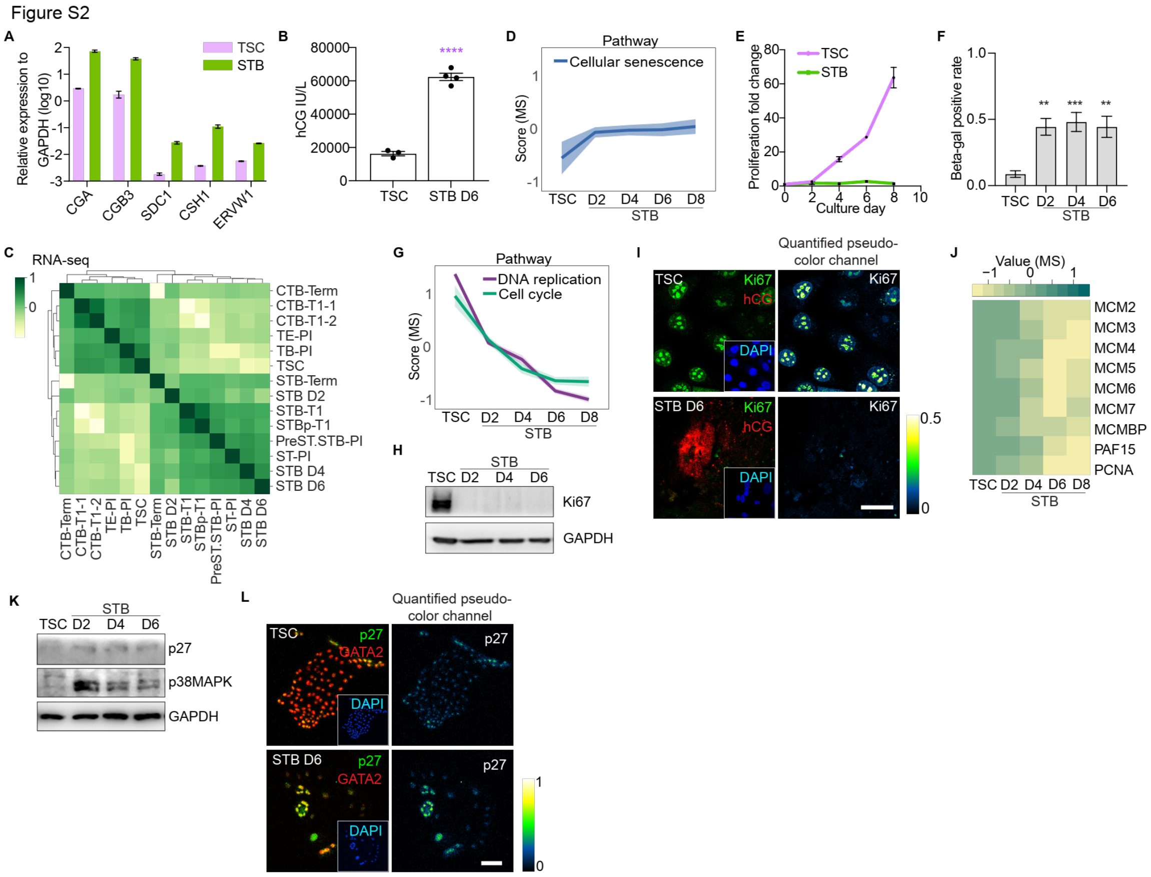

Figure S2. The development of hTSCs to STBs *in vitro* has the major senescence hallmarks, including cell cycle arrest, similar to STBs *in vivo*. **A**. qPCR of genes of markers CGA, CGB3, SDC1, CSH1 and ERVW1 in hTSCs and STBs; **B**. ELISA detection of higher β-hCG secretion in STBs than hTSCs; **C**. Kinship of *in vitro* derived STBs with different cell types from several placenta developmental stages (PI: peri-implantation, Pre: pre-implantation, T1: first-trimester, Term: term trimester). *In vitro* STBs are close to STBs from the PI and the T1 stages. hTSCs

had the greatest kinship with peri-implantation TE/TB; **D**. Analysis of mass spectrometry (MS) data indicated an increasing senescence score from hTSCs to STBs of D2, D4, D6, D8; **E**. The fold change of hTSCs and STBs proliferation rate over culture days (day 0, 2, 4, 6, 8); **F**. More STBs (D2, D4, D6) are positively stained for SA-β-gal than hTSCs; **G**. Line chart of Mass Spectrometry pathway analysis of DNA replication and cell cycle. From hTSCs to STBs (D2, D4, D6, D8), the scores of both pathways are rapidly decreased; **H**. Western blot of the proliferation marker Ki67 in hTSCs and STBs (D2, D4, D6); **I**. Representative immunostaining images of Ki67 in hTSCs and STB D6 cells; **J**. Mass Spectrometry analysis of proliferation markers (PCNA, PCNA cofactor PAF15, and MCMs) along hTSCs to STBs (D2, D4, D6, D8); **K**. Western blot analysis of p27 and p38MAPK in hTSCs and STBs (D2, D4, D6); **L**. Representative immunostaining images of p27 in hTSCs and STBs (D6). Data are mean ± SEM. The t-test is used in statistical analysis. No significance (ns), $P > 0.05$; * $P \leq 0.05$, ** $P \leq 0.01$; *** $P \leq 0.001$; **** $P \leq 0.0001$. All experiments have been independently repeated three times. Scale bars, 10 μm.

Figure S3

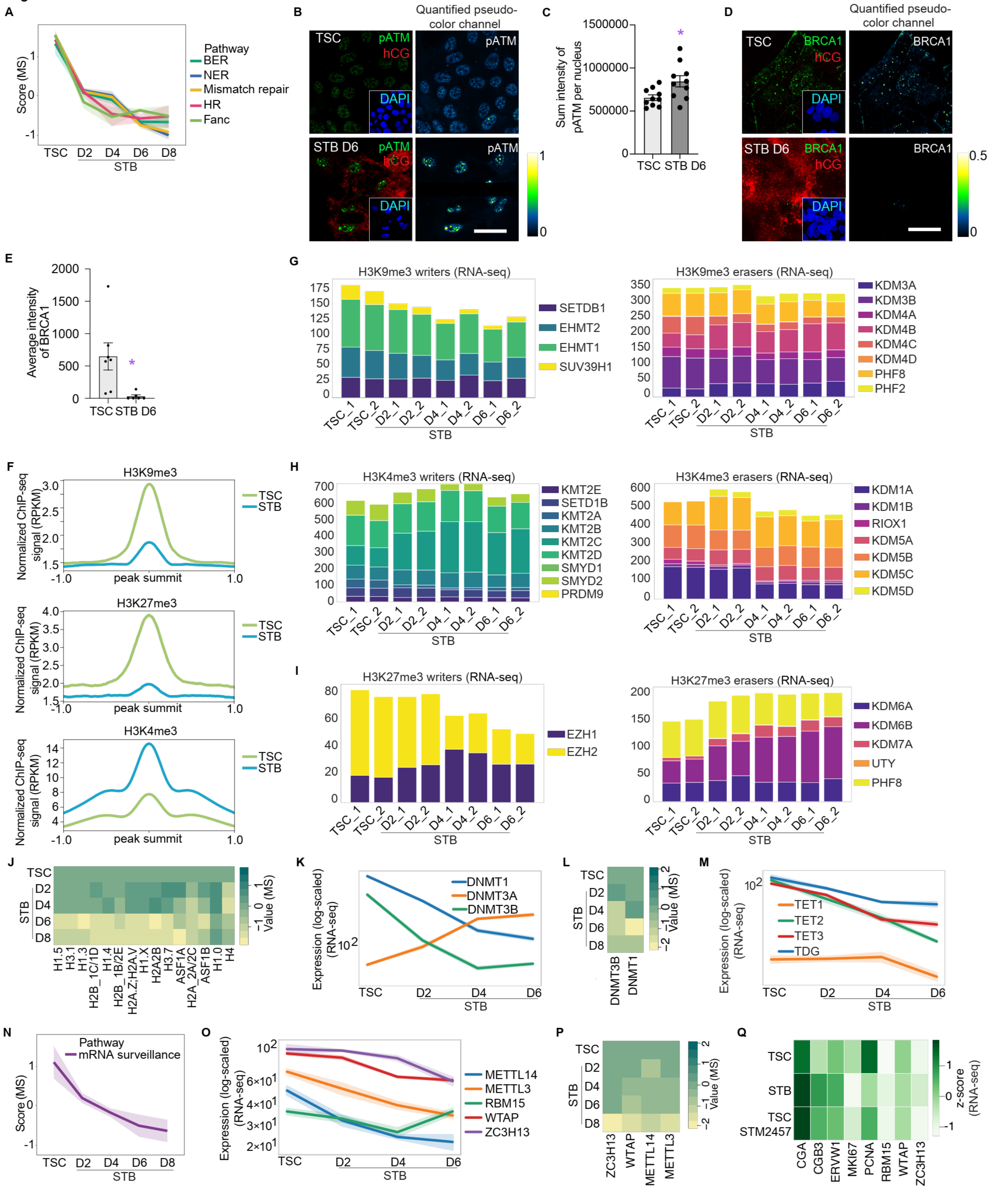

Figure S3. DNA damage repair decline, genome instability and epigenetic alterations in STBs. **A**. Proteomics analysis of DNA damage repair pathways (BER, NER, mismatch repair, HR, and Fanc); **B**. Representative immunostaining images for activated phospho-ATM (pATM) in hTSCs and STBs (D6); **C**. Sum intensity of activated pATM in hTSCs and STBs (D6); **D**. Representative immunostaining images of BRCA1 in hTSCs and STBs (D6); **E**. Immunostaining intensity of BRCA1 in hTSCs and STBs (D6); **F**. CHIP-seq data analysis showed normalized H3K9me3, H3K27me3, and H3K4me3 signaling in hTSCs and STBs; **G**

& **H** & **I**. Stacked bar plots of genes encoding writers and erasers of H3K9me3, H3K4me3, and H3K27me3, respectively; **J**. Heatmap of cononical histones changes from hTSCs to STBs (D2, D4, D6, D8) in proteomics analysis; **K**. Line chart of expression of genes encoding DNA methyltransferases DNMT1, DNMT3A, and DNMT3B along hTSCs to STBs; **L**. Proteomics analysis of DNMT1 and DNMT3B in hTSCs and STBs; **M**. Line chart of expression of genes encoding DNA demethyltransferases TET1, TET2, TET3, and TDG along hTSCs to STBs; **N**. A marked decreasing score for mRNA surveillance in hTSC-STB system at protein level; **O**. Transcriptomical line chart of genes encoding RNA methylation related regulator METTL3, METTL14, RBM15, WTAP, and ZC3H13; **P**. Heatmap of RNA methylation related regulators METTL3, METTL14, WTAP, and ZC3H13 in Mass Spectrometry analysis. **Q.** Expression levels of differentiation markers (CGA, CGB3, and ERVW1) and proliferation markers (MKI67, PCNA) in hTSCs, STBs and METTL3 inhibitor STM2457-treated hTSCs. Data are mean $\pm$ SEM. The t-test is used in the statistical analysis. No significance (ns), $P > 0.05$; * $P \leq 0.05$, ** $P \leq 0.01$; *** $P \leq 0.001$; **** $P \leq 0.0001$. All experiments have been independently repeated three times. Scale bars, 10 μm.

Figure S4

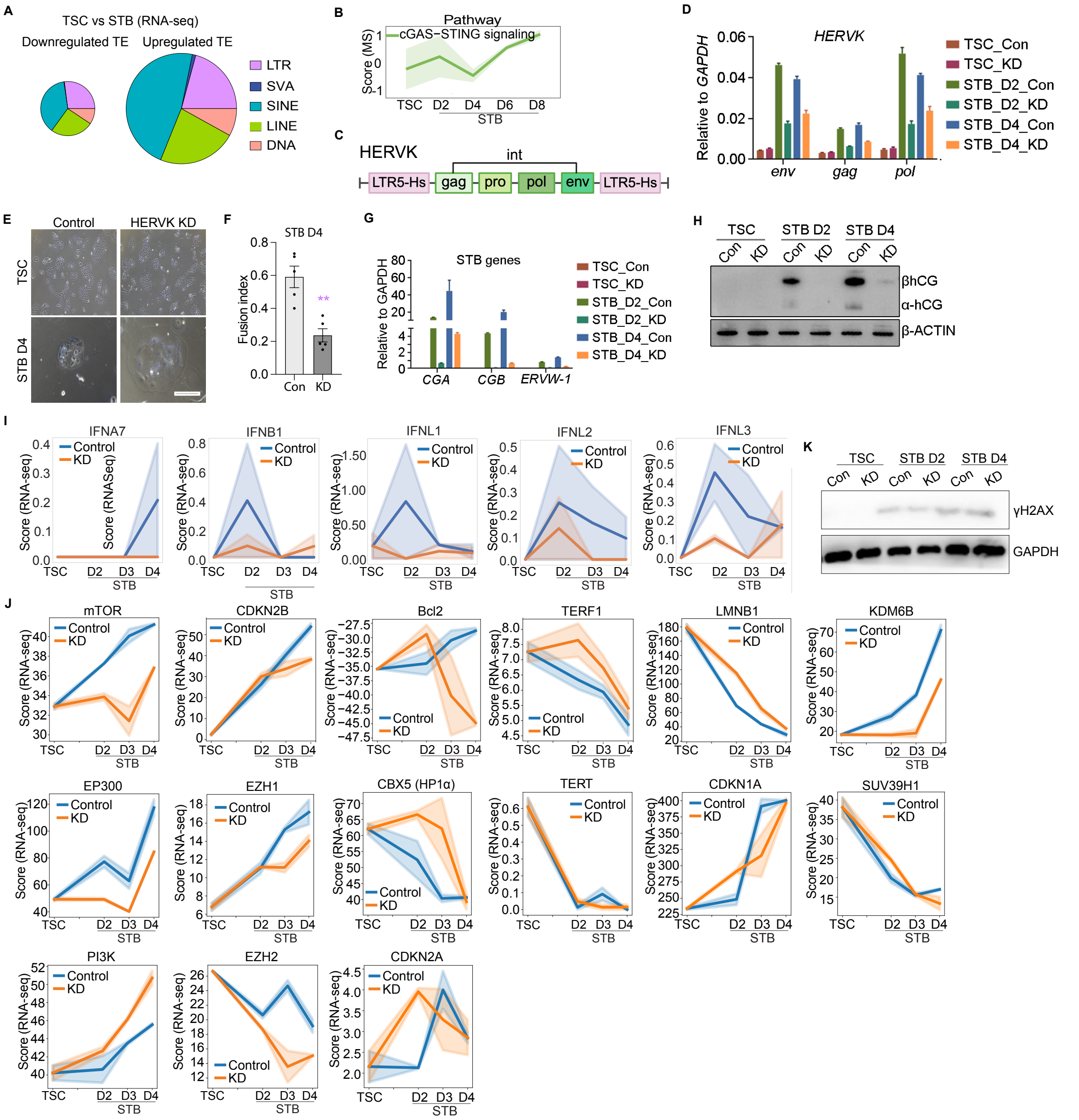

Figure S4. Reactivation of transposon elements (TEs) in STBs is a driving senescence hallmark. **A**. The TE class distribution of differentially expressed TE loci in STBs (D2) versus hTSCs. The size of the circle is proportional to the number of differentially expressed TE loci; **B**. Line chart score of cGAS-STING signalling pathway along hTSCs to STBs (D2, D4, D6, D8) in proteomics analysis; **C**. Schematic diagram of HERVK; **D**. RT-qPCR analysis of HERVK components (env, gag, pol) in hTSCs and STBs (D2, D4) in the control (Con, no dox) and the LTR5_Hs-KD (dox-treated) groups; **E**. Morphology of the control and LTR5_Hs -KD hTSCs and STBs (D4); **F.** Bar chart showed Fusion index of STB (D4) cells in the Con and the LTR5_Hs-KD groups; **G**. RT-qPCR analysis of STB genes (CGA, CGB, ERVW-1) in hTSCs and STBs of the Con and the LTR5_Hs-KD groups; **H**. Western blot analysis of α-hCG (CGA)

and β-hCG (CGB) in LTR5_Hs -KD STBs (D2 and D4); **I**. Transcriptomics analysis of genes encoding interferons (IFNLs) in hTSCs to STBs in the Con and the LTR5_Hs-KD STB groups, respectively; **J**. Line charts showing expression of ageing associated genes (log scale, y-axis) across hTSCs to STBs (D2, D3, D4) (x-axis). The Control is in blue and LTR5_Hs-KD is in orange. Each point on the line reflects the average value between two replicates, while their standard errors are reflected by the shade of the line; **K**. Western blot of γH2AX level in hTSCs and STBs (D2, D4) of the Con and the LTR5_Hs-KD groups. All experiments have been independently repeated three times. Scale bars, 10 μm.

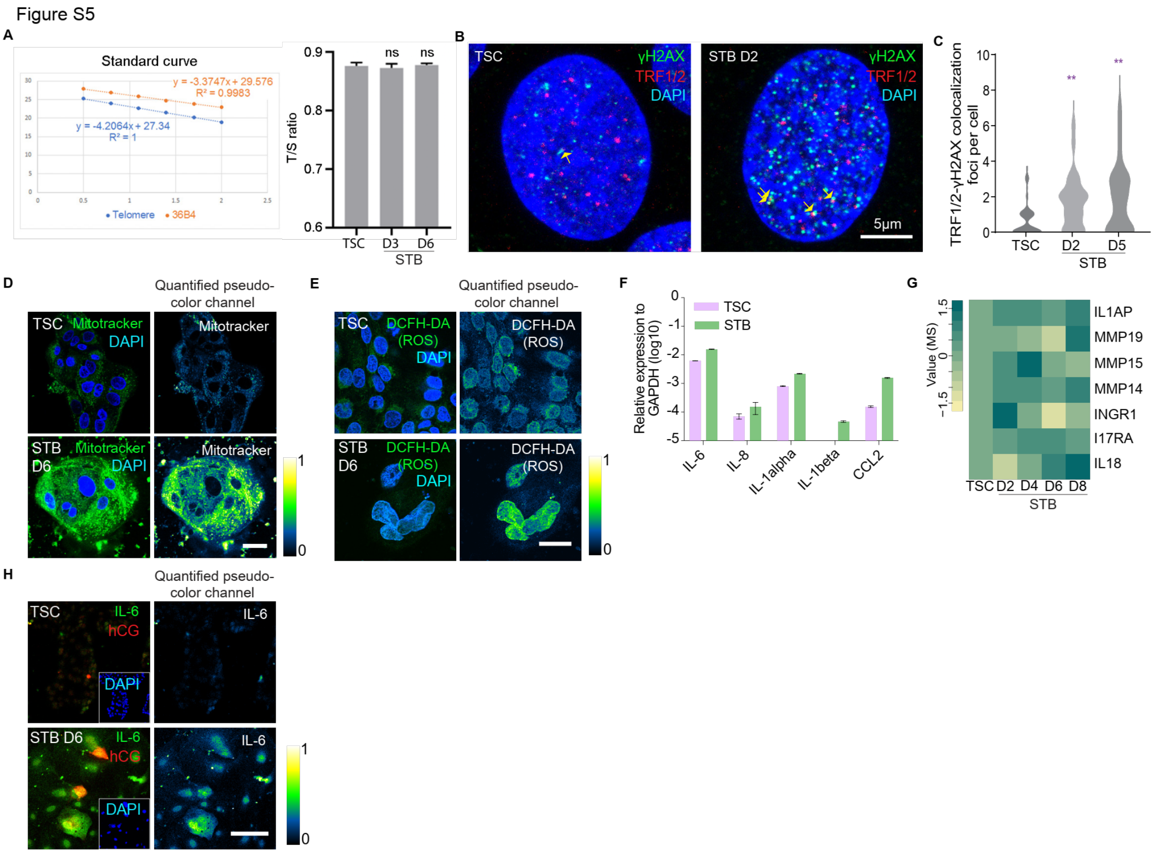

Figure S5. STBs generated from hTSCs have organelle dysfunction and senescence-associated secretory phenotypes (SASPs). **A**. No statistically significant changes in telomere length in hTSCs and STBs (D3, D6); **B**. Representative immunostaining images of γH2AX and TRF1/2 nuclear co-localization in hTSCs and STBs (D2). The yellow arrows point to representative colocalization sites; **C**. Statistically significant more colocalization of γH2AX and TRF1/2 in STBs (D2, D5) than hTSCs; **D**. Mitotracker staining for mitochondria in hTSCs and STBs (D6); **E**. DCFH-DA staining for ROS and its localization in hTSCs and STBs (D6); **F**. RT-qPCR of genes encoding for SASP factors (IL-6, IL-8, IL-1alpha, IL-1beta, CCL2) in hTSCs and STBs (D6); **G**. Mass Spectrometry detection of SASP factors in hTSC and STBs (D2, D4, D6, and

D8); **H**. The representative co-immunostaining images of IL-6 with hCG in hTSCs and STBs. Data are mean ± SEM. One-way ANOVA with Dunnett's test was used in statistical analysis. No significance (ns), $P > 0.05$; * $P \leq 0.05$, ** $P \leq 0.01$; *** $P \leq 0.001$; **** $P \leq 0.0001$. All experiments have been independently repeated three times. Scale bars, 10 μm.

Figure S6

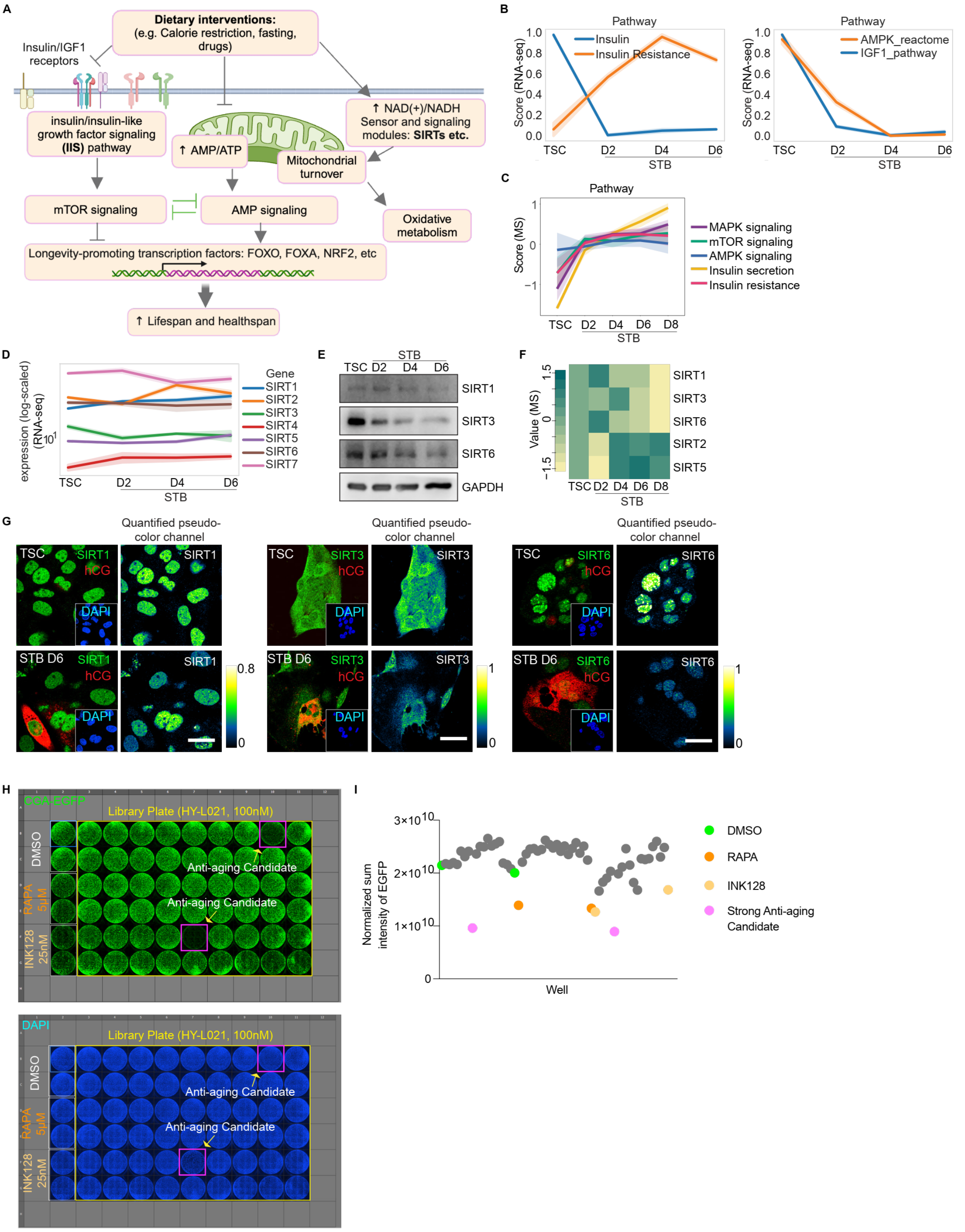

Figure S6. Calorie restriction-related aging pathways in the hTSC-STB system. **A**. Diagram

of correlation of calorie restriction and longevity; **B.** Line chart of gene expression scores of nutrient-related pathways (insulin, insulin resistance, IGF1, AMPK) in hTSCs and STBs; **C**. Proteomics score trends of nutrient-related pathways (insulin secretion, insulin resistance pathway, mTOR signalling, AMPK, and MAPK signalling) in hTSCs and STBs (D2, D4, D6, D8); **D**. Line chart showing gene expression of SIRT family members (SIRT1, SIRT2, SIRT3, SIRT4, SIRT5, SIRT6, SIRT7) in hTSCs and STBs; **E**. Western blot detection of SIRT1, SIRT3, and SIRT6 in hTSCs and STBs (D2, D4, D6); **F**. Mass spectrometry data analysis of SIRTs in hTSCs and STBs (D2, D4, D6, D8); **G**. Representative immunostaining images of SIRT1, SIRT3 and SIRT6 in hTSCs and STBs (D6); **H**. An example of a screened 96 well library plate. Rapamycin and INK128 were used as the positive controls. The Magenta rectangle marked the stronger potential anti-aging candidate wells; **I**. Quantification of EGFP signals of the 96 well plate in **H**. The sum intensity was normalized, referring to DAPI signals. All experiments have been independently repeated three times. Scale bars, 10 μm.
